# Naringenin prevents TGF-β1 secretion from breast cancer and suppresses pulmonary metastasis by inhibiting PKC activation

**DOI:** 10.1186/s13058-016-0698-0

**Published:** 2016-04-01

**Authors:** Fayun Zhang, Wenjuan Dong, Wenfeng Zeng, Lei Zhang, Chao Zhang, Yuqi Qiu, Luoyang Wang, Xiaozhe Yin, Chunling Zhang, Wei Liang

**Affiliations:** Protein & Peptide Pharmaceutical Laboratory, Institute of Biophysics, Chinese Academy of Sciences, Beijing, 100101 China; Department of Gynecology, The First Affiliated Hospital of Yangtze University, Jingzhou, Hubei 434000 China

**Keywords:** Naringenin, TGF-β1 secretion, Breast cancer metastasis, PKC activation

## Abstract

**Background:**

Targeting the TGF-β1 pathway for breast cancer metastasis therapy has become an attractive strategy. We have previously demonstrated that naringenin significantly reduced TGF-β1 levels in bleomycin-induced lung fibrosis and effectively prevented pulmonary metastases of tumors. This raised the question of whether naringenin can block TGF-β1 secretion from breast cancer cells and inhibit their pulmonary metastasis.

**Methods:**

We transduced a lentiviral vector encoding the mouse *Tgf-β1* gene into mouse breast carcinoma (4T1-Luc2) cells and inoculated the transformant cells (4T1/TGF-β1) into the fourth primary fat pat of Balb/c mice. Pulmonary metastases derived from the primary tumors were monitored using bioluminescent imaging. Spleens, lungs and serum (n = 18–20 per treatment group) were analyzed for immune cell activity and TGF-β1 level. The mechanism whereby naringenin decreases TGF-β1 secretion from breast cancer cells was investigated at different levels, including *Tgf-β1* transcription, mRNA stability, translation, and extracellular release.

**Results:**

In contrast to the null-vector control (4T1/RFP) tumors, extensive pulmonary metastases derived from 4T1/TGF-β1 tumors were observed. Administration of the TGF-β1 blocking antibody 1D11 or naringenin showed an inhibition of pulmonary metastasis for both 4T1/TGF-β1 tumors and 4T1/RFP tumors, resulting in increased survival of the mice. Compared with 4T1/RFP bearing mice, systemic immunosuppression in 4T1/TGF-β1 bearing mice was observed, represented by a higher proportion of regulatory T cells and myeloid-derived suppressor cells and a lower proportion of activated T cells and *INFγ* expression in CD8^+^ T cells. These metrics were improved by administration of 1D11 or naringenin. However, compared with 1D11, which neutralized secreted TGF-β1 but did not affect intracellular TGF-β1 levels, naringenin reduced the secretion of TGF-β1 from the cells, leading to an accumulation of intracellular TGF-β1. Further experiments revealed that naringenin had no effect on *Tgf-β1* transcription, mRNA decay or protein translation, but prevented TGF-β1 transport from the trans-Golgi network by inhibiting PKC activity.

**Conclusions:**

Naringenin blocks TGF-β1 trafficking from the trans-Golgi network by suppressing PKC activity, resulting in a reduction of TGF-β1 secretion from breast cancer cells. This finding suggests that naringenin may be an attractive therapeutic candidate for TGF-β1 related diseases.

**Electronic supplementary material:**

The online version of this article (doi:10.1186/s13058-016-0698-0) contains supplementary material, which is available to authorized users.

## Background

Breast cancer is the most common malignancy in female cancer patients, accounting for 23 % of all new cancer cases worldwide. Metastasis from the primary breast tumor is the leading cause of mortality among these patients [1, 2]. An association between elevated cellular and plasma transforming growth factor (TGF)-β1 levels and increased breast cancer metastasis has been reported previously [3–7]. Secreted TGF-β1 promotes metastasis via transforming T cells to regulatory T cells (Tregs), which facilitates the escape of cancer cells from host immune responses [8], and/or by inducing cancer cells to undergo epithelial to mesenchymal transition (EMT) [9]. The proliferation of cancer cells increases TGF-β1 secretion, leading to a more metastatic phenotype [10]. Clinical studies have reported a link between the presence of a tumor and an increase of CD4^+^CD25^+^ cells in the blood [11–13]. Tregs constitutively express *Foxp3*, which is the master regulator that mediates the immunosuppressive function of Tregs [14–16].

Small molecule TGF-β1 inhibitors are a feasible approach for breast cancer metastasis prevention and therapy. TGF-β1 blocking antibodies, short hairpin RNA of TGF-β1, and inhibitors of TGF-β1 receptors have been shown to prevent tumor development and metastasis [17–19]. However, complete inhibition of TGF-β1 signaling by receptor inhibitors or antibodies showed serious adverse effects, such as gastrointestinal and skin-related events [20], thrombocytopenia, and keratoacanthomas/squamous cell carcinoma [21].

TGF-β proteins are synthesized as inactive precursor proteins with an amino-terminal prodomain followed by the carboxyl-terminal mature ligand. Precursor proteins dimerize in the endoplasmic reticulum (ER), where they are cleaved by serine proteases. Following processing, they are transported through the trans-Golgi network (TGN) to the cell surface for release and to the lysosome for degradation [22–24]. Inhibiting the proteins that target TGF-β for secretion may be a new strategy to block signal transduction of the TGF-β pathway.

Naringenin, the predominant flavanone in grapefruit, is reported to possess a broad range of pharmacological activities, such as antioxidant, anti-inflammatory, carbohydrate metabolism promotion, and modulating the immune system [25]. Our previous study showed that naringenin reduced TGF-β1 levels in fibrotic microenvironments and prevented fibrosis-induced lung metastasis [26], and that naringenin inhibited TGF-β1-induced migration and invasion of pancreatic cancer cells [27]. In the context of fibrosis-induced lung metastasis, TGF-β1 could be derived from various cell types, including cancer cells, fibroblasts, macrophages, epithelial cells, and immune cells. To investigate the role of breast tumor-derived TGF-β1 for metastasis, and whether naringenin can suppress metastasis by blocking TGF-β1 secretion from breast tumor cells, we constructed a *Tgf-β1* overexpressing breast tumor cell line (4T1/TGF-β1) and examined its metastatic potential in both in vitro and in vivo models. Our data demonstrated that naringenin effectively reduced TGF-β1 release and suppressed tumor cell migration and pulmonary metastasis. Unexpectedly, naringenin prevented TGF-β1 secretion by a post-translational mechanism, which differs from TGF-β neutralizing antibodies and TGF-β receptor antagonists. The results of this study may provide a novel therapeutic approach for intervention of TGF-β signaling pathway-related diseases and disorders. More importantly, our study reveals that targeting the intracellular trafficking machinery of cytokines may be an attractive strategy for developing new anti-cytokine therapies.

## Methods

### Cell lines and materials

The murine breast cancer cell line 4T1 was purchased from American Type Culture Collection (Manassas, VA, USA). 4T1 cells, the vector control (4T1/RFP), and TGF-β1-overexpressing 4T1-Luc2 cells (4T1/TGF-β1) were cultured in RPMI 1640 medium. 1D11 antibody was from eBioscience Tech (San Diego, CA, USA). Naringenin was purchased from Shanxi Huike Botanical Development Co. (Xi'an, China).

### Generation of 4T1/TGF-β1 transformants

Human growth hormone signal sequence was synthesized and fused with the full-length mouse *Tgf-β1* gene using PCR. The hybrid gene of human growth hormone signal sequence and mouse *Tgf-β1* was then ligated into pSin-EF2-Oct4-Pur plasmid (Addgene, Cambridge, MA, USA) to replace Oct4 with *Spe*I and *Eco*RI cloning sites at the 5′ and 3′ termini, respectively. RFP from the pmRFP-C1 plasmid (Clontech, Mountain View, CA, USA) was then cloned into the pSin-EF2-TGFβ1-Pur plasmid to replace puromycin. The generated *Tgf-β1* overexpression vectors were then enveloped in 293T cells. The medium containing the packaged virus was used to infect 4T1-Luc2 breast cancer cells (PerkinElmer, Waltham, MA, USA) to generate 4T1/TGF-β1 transformants. The control transformants, 4T1/RFP cells, were generated using the vector without *Tgf-β1 * gene, following the same procedures. 4T1/RFP and 4T1/TGF-β1 transformants were then sorted by flow cytometry with excitation/emission of 578/603 nm.

### In vivo breast cancer metastasis experiments

Four-week-old female Balb/c mice were purchased from Weitonglihua Tech. (Beijing, China) and housed in the Animal Care Facility of the Institute of Biophysics, Chinese Academy of Sciences, China. All animal protocols used for this study were approved by the Institutional Animal Care and Use Committee. The fourth mammary fat pads of Balb/c mice were injected with 2 × 10^4^ 4T1/RFP or 4T1/TGF-β1 cells. Beginning on the same day, the mice were administered 200 mg/kg naringenin once daily for 30 days (suspension in 1 % sodium carboxyl methyl cellulose (CMCNa)) or 5 mg/kg 1D11 antibody (dilution in phosphate-buffered saline buffer) twice a week for 3 weeks. The primary tumor and lung metastases were imaged by bioluminescence using the IVIS Spectrum In Vivo Imaging System (Xenogen, Caliper Life Science, PerkinElmer, Hopkinton, MA, USA ) as described previously [28]. Briefly, tumor-bearing mice were given intraperitoneal injections with 150 mg/kg luciferin and the lung areas were imaged. To avoid the bioluminescence from the primary tumor, primary tumors were wrapped with light-proof bags. After 4 weeks of primary tumor growth, mice were sacrificed after intraperitoneal injection of luciferin for 15 minutes and the lungs were collected for imaging or weighing to determine the amount of metastases. The weight of tumor burden in the lung was calculated by subtracting the mean weight of normal lungs (0.15 g) from the weight of the lungs with metastatic tumors; primary tumors were also isolated and weighted. After treatment, the lungs and spleens of each mouse at day 14 and day 28 were dissected for quantitative PCR (qPCR), T-cell immunity assays, and immunohistochemistry. The levels of activated TGF-β1 in the primary tumors, spleens, lungs, and serum were detected by enzyme-linked immunosorbent assay (ELISA). The bioluminescence imaging, qPCR [29], T-cell immunity assays, immunohistochemistry, and TGF-β1 ELISA were performed as described previously [26] (details in Additional file 1). The survival endpoint of mice was defined as the day of the mouse’s death.

### T-cell activity assays

In vivo assays of T-cell activation were performed as described previously [26]. Briefly, isolated lymphocytes from the spleen and lung were surface-stained with fluorochrome-conjugated anti-CD4, anti-CD44, and anti-CD62L antibodies. To assay myeloid-derived suppressor cells (MDSC), isolated splenocytes or purified T cells from the lungs were stained with fluorochrome-conjugated anti-Gr-1 and anti-CD11b antibodies. To analyze the production of interferon gamma (IFNγ) in CD8^+^ T cells, isolated splenocytes were stimulated with 25 ng/ml phorbol 12-myristate 13-acetate (Sigma, St. Louis, MO, USA) and 500 ng/ml ionomycin (Sigma) for 5 hours. After further incubation with Brefeldin A Solution (eBioscience) for 3 hours, the stimulated cells were surface-stained with anti-CD8 antibodies, fixed and permeabilized, and then stained with anti-IFNγ antibody.

For Treg assays, the isolated splenocytes or purified T cells from lungs were separated using CD4 MACS beads (Miltenyi Biotec, Cologne, Germany). The separated CD4^+^ T cells were cultured in the supernatants with anti-CD3 antibody (3 μg/ml) and anti-CD28 antibody (1 μg/ml) for 72 hours. The cells were surface-stained with anti-CD4 and anti-CD25 antibodies, fixed and permeabilized, and then stained with anti-Foxp3 antibody. The stained cells were analyzed by flow cytometry using a FACSCalibur system with CellQuest software (BD Biosciences, San Jose, CA, USA).

### Transfer of T cells and 4T1/TGF-β1 cancer cells into nude mice

T cells were isolated from the spleen of normal (control group) or 4T1/TGF-β1 tumor-bearing Balb/c mice with naringenin or 1 % CMCNa treatment. After red blood cells were lysed by ACK hypotonic lysis solution (Sigma), T cells were purified using a pan T-cell isolation kit (Miltenyi Biotech) in accordance with the manufacturer’s protocol. Balb/c nude mice were then intravenously injected with 5 × 10^5^ of these purified T cells mixed with 5 × 10^3^ 4T1/TGF-β1 tumor cells (100:1 ratio of T cells to 4T1/TGF-β1 tumor cells). T cells with tumor cells were transferred once a week for 3 weeks. The transfer of the mixed cells to nude mice was performed as described previously [30]. The bioluminescence of lung metastasis in nude mice on day 28 was imaged using the IVIS system as described previously [28] (details in Additional file 1).

### Confocal imaging

Cellular localization of TGF-β1 in 4T1 cells with different treatments was determined by immunofluorescence staining with Alexa 633-labeled secondary IgG antibody after anti-TGF-β1 antibody binding. The TGN was stained with Alexa 488-labeled secondary IgG antibody after anti-TGN46 antibody binding. The nuclei were stained with Hoechst 33342. Stained cells were observed using confocal microscopy as described [31] previously (details in Additional file 1).

### Statistical analysis

All in vivo experiments included at least five mice per group. Results are presented as median ± interquartile (IQ) range or median ± standard error (SE). Analysis of results containing two groups was carried out using the Student *t* test, assuming unequal variance. Two-way analysis of variance was performed to determine the variations of bioluminescent photons between the control and TGF-β1 overexpression groups. The survival curves were analyzed using log-rank (Mantel–Cox test) analysis. *P* <0.05 was considered statistically significant.

## Results

### TGF-β1 overproduction in 4T1 tumor cells promotes pulmonary metastasis

**Fig. 1:**
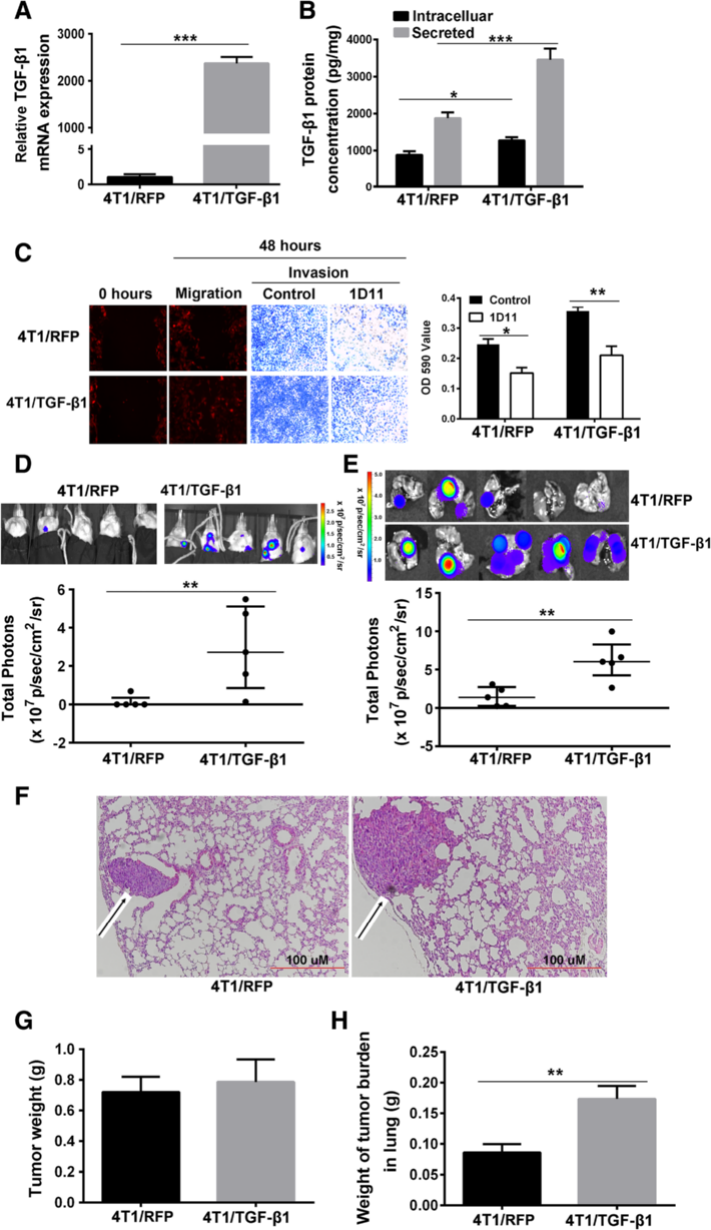
TGF-β1 overproduction in 4T1 cells promotes pulmonary metastasis. **a** *Tgf-β1* mRNA relative expression level in cultured 4T1/RFP cells and 4T1/TGF-β1 cells, *P* <0.001. **b** Intracellular and secreted TGF-β1 protein concentration in cultured 4T1/RFP cells and 4T1/TGF-β1 cells. **c** Migration and invasion of cultured 4T1/RFP cells and 4T1/TGF-β1 cells treated with 1D11 were observed under the fluorescent microscopy. Migration and invasion were detected by wound healing and transwell respectively after cells were treated with 1D11 for 48 hours (*left*). The invaded 4T1/RFP and 4T1/TGF-β1 cells treated with 1D11 were dissolved in DMSO and OD590 values were determined using the multimode reader (*right*). **d** Bioluminescence imaging of 4T1 breast cancer lung metastasis on day 28 in vivo using IVIS, *P* = 0.023. (Median ± IQ range.) **e** Bioluminescence imaging of breast cancer metastasis in the dissected lungs. *P* = 0.006. (Median ± IQ range.) **f** Histological imaging of lung tissue sections of mice bearing 4T1/RFP tumors or 4T1/TGF-β1 tumors on day 28. Hematoxylin and eosin staining was carried out in 5 μm sections of paraffin-embedded lung tissues. *Black arrow* indicates metastatic tumor. **g** Primary tumor weight of mice bearing 4T1/RFP tumor or 4T1/TGF-β1 tumor on day 28. **h** Weights of tumor burden in lungs of mice bearing 4T1/RFP tumor and 4T1/TGF-β1 tumor on day 28. Results are representative of at least three experiments. *P <0.05, **P <0.01, ***P <0.001. Error bars indicate SE. *TGF* transforming growth factor

To investigate the role of TGF-β1 overexpression in 4T1 cells in pulmonary metastasis, we constructed a lentiviral vector that expressed a hybrid construct containing human growth hormone signal sequence and murine *Tgf-β1*, which enabled the transduced cells to secrete mature TGF-β1 (4T1/TGF-β1). Compared with the null-vector control cells (4T1/RFP), 4T1/TGF-β1 cells showed increased expression of *Tgf-β1* mRNA (2332-fold increase; *P* <0.001), as well as increased cellular and secreted TGF-β1 (*P* = 0.05 and *P* = 0.01, respectively) (Fig. 1a, b). The 4T1/TGF-β1 cells also showed increased migration and invasion compared with 4T1/RFP cells in vitro (Fig. 1c). However, cellular invasion of both 4T1/RFP and 4T1/TGF-β1 was inhibited by addition of TGF-β1 neutralizing antibody (1D11) to the medium (Fig. 1c). Furthermore, we quantitated TGF-β1 secretion in several human breast cancer cell lines, and found that secreted TGF-β1 levels were positively related to invasiveness of the cancer cells (Additional file 2: Figure S1).

**Fig. 2:**
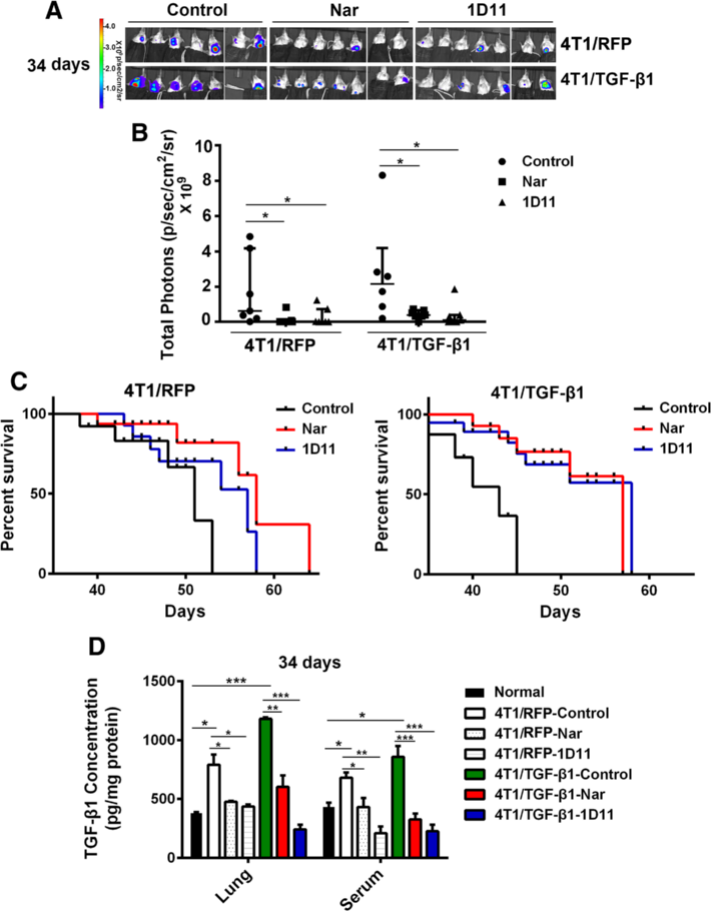
Naringenin (*Nar*) prevents pulmonary metastasis derived from the primary breast tumor. **a** Bioluminescence imaging of pulmonary metastasis in vivo. Mice bearing 4T1 tumor were treated with Nar or 1D11 for 34 days and then were imaged with bags to avoid the bioluminescence from primary tumor. **b** Bioluminescent photons of the pulmonary area from the mice treated with Nar or 1D11 antibodies for 34 days measured using the IVIS system (median ± IQ range). **c** Survival of mice bearing 4T1/RFP tumor (*left*) or 4T1/TGF-β1 (*right*) tumor after treatment with Nar or 1D11 antibodies. **d** TGF-β1 concentration in lung homogenates and serum. Results are representative of three experiments. *P <0.05, **P <0.01, ***P <0.001. Error bars indicate SE. *TGF* transforming growth factor

We next investigated whether *Tgf-β1* overexpression led to extensive pulmonary metastases*.* 4T1/TGF-β1 cells and 4T1/RFP cells were injected into the fourth breast pads of mice, and pulmonary metastases were determined at day 28 after injection. The bioluminescence images showed that all of the mice bearing 4T1/TGF-β1 tumors had pulmonary metastases with brighter bioluminescence than the mice bearing 4T1/RFP tumors, in which only one mouse had detectable bioluminescence before the dissected lungs were exposed (for the in vivo lung imaging, *P* = 0.023; for the dissected lung imaging, *P* = 0.006) (Fig. 1d, e). The histological sections of lungs from mice bearing 4T1/TGF-β1 tumors showed larger metastatic lesions than those from mice bearing 4T1/RFP tumors (Fig. 1f). The tumor burden weight in removed lungs from mice bearing 4T1/TGF-β1 tumors was greater than that of lungs from mice bearing 4T1/RFP tumors (*P* <0.001) (Fig. 1h), while the tumor weight at the primary site did not show a significant difference between the mice bearing 4T1/RFP cells and the mice bearing 4T1/TGF-β1 cells (*P* = 0.43) (Fig. 1g and Additional file 3: Figure S2). The extensive pulmonary metastases in the 4T1/TGF-β1 tumor-bearing mice could lead to a decreased life span. Indeed, the survival analysis showed that all of the 4T1/TGF-β1 tumor-bearing mice without any treatment died at day 45 after tumor cell injection, while the 4T1/RFP tumor-bearing mice without treatment displayed an increased life span (*P* = 0.007) (Fig. 2c).

### Naringenin inhibits pulmonary metastasis from the primary breast tumor

As shown in Fig. 2, naringenin administration had an inhibitory effect on pulmonary metastasis at day 34 (similar inhibitory effects on pulmonary metastasis at day 24 shown in Additional file 3: Figure S2A) and prolonged survival to levels comparable with 1D11 antibody administration in 4T1/TGF-β1 tumor-bearing mice, but better than 1D11 in 4T1/RFP tumor-bearing mice (for survival in 4T1/RFP: naringenin vs control, *P* = 0.032 and 1D11 vs control, *P* = 0.19; for survival in 4T1/TGF-β1: naringenin vs control, *P* = 0.012 and 1D11 vs control, *P* = 0.021) (Fig. 2a–c). In contrast to the untreated control, naringenin and 1D11 treatments decreased the incidence of pulmonary metastasis in tumor-bearing mice on day 24 and day 34 (for 4T1/TGF-β1 tumor-bearing mice on day 24: 57 % (4/7) in control group, 14 % (1/7) in naringenin group, and 29 % (2/7) in 1D11 group; for 4T1/RFP tumor-bearing mice on day 34: 100 % (7/7) in control group, 29 % (2/7) in naringenin group, and 43 % (3/7) in 1D11 group) (Additional file 4: Table S1). The growth of the primary tumor was not significantly affected by naringenin treatment or by 1D11 treatment compared with the untreated control mice (Additional file 3: Figure S2B, C). The reduction of pulmonary metastasis by naringenin and 1D11 treatments could result from lower levels of activated TGF-β1 circulating in the serum of tumor-bearing mice. Therefore, we determined the levels of the activated TGF-β1 in the serum and in the lung on day 34. Compared with normal mice, the activated TGF-β1 concentration in lung tissues and circulating serum was significantly elevated both in 4T1/RFP and 4T1/TGF-β1 tumor-bearing mice. As expected, naringenin and 1D11 treatments reduced active TGF-β1 levels both in the lung and serum (Fig. 2d).

To ascertain the role of tumor-secreted TGF-β1 in promoting pulmonary metastasis, we determined levels of the activated form of TGF-β1 in the tumor, spleen, lung, and serum on days 14 and 28 after tumor cell implantation. Activated TGF-β1 was increased in all samples, including tumor, lung, and serum from tumor-bearing mice compared with tumor-free mice (*P* <0.05). Naringenin treatment significantly reduced activated TGF-β1 levels in the tissues and serum in tumor-bearing mice (*P* <0.05) (Additional file 5: Figure S3A, B). Tumor-derived TGF-β1 has been showed to induce the expression of *Foxp3*, which is a firm link between TGF-β1 levels and Tregs [32, 33]. In agreement with these studies, an increase of *Foxp3* expression was observed in the lungs of tumor-bearing mice, particularly in mice bearing 4T1/TGF-β1 tumors. Naringenin administration reduced *Foxp3* expression in the lungs of tumor-bearing mice (Additional file 5: Figure S3D). The immunohistochemical images of the lungs on day 14 and day 28 after tumor injection showed a histologic disruption of lung structure and an increase of TGF-β1 staining. Naringenin treatment decreased TGF-β1 staining and restored normal lung architecture (Additional file 5: Figure S3C).

### Naringenin modulates the immunosuppressive environment in tumor-bearing mice

**Fig. 3:**
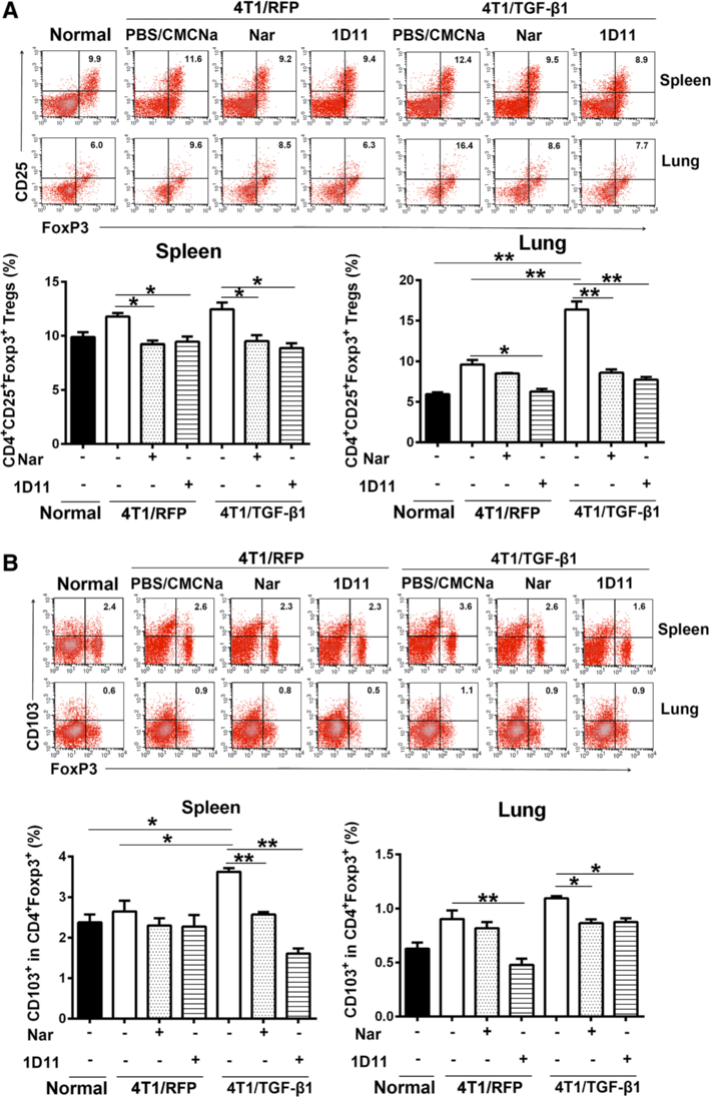
Naringenin (*Nar*) reduces the population of Tregs in spleens and lungs. **a** Flow cytometry analysis of the percentage of CD4^+^CD25^+^Foxp3^+^ cells (Tregs) in lymphocytes purified from spleen and lung tissues. **b** Flow cytometry analysis of the percentage of CD103^+^CD4^+^ Foxp3^+^ cells in lymphocytes purified from spleen and lung tissues. Column charts show the mean percentage obtained from three experiments. Percentage gated cells are shown. Results are representative of three or four experiments. *P <0.05, **P <0.01, ***P <0.001. Error bars indicate SE. *CMCNa* sodium carboxyl methyl cellulose, *PBS* phosphate-buffered saline, *TGF* transforming growth factor

The elevated TGF-β1 levels were accompanied by increased *Foxp3* expression in the lungs of 4T1/TGF-β1 tumor-bearing mice, which aroused our interest in the relationship between TGF-β1 production and the Treg population. Because previous studies have revealed that tumor-derived TGF-β1 mediates the conversion of CD4^+^Foxp3^+^ Tregs [34], we measured the percentage of CD4^+^CD25^+^Foxp3^+^ Tregs and CD103^+^CD4^+^Foxp3^+^ cells in lymphocytes from the spleens and lungs of tumor-bearing mice. The expression of *CD103* in lymphoid CD4^+^Foxp3^+^ cells was reported to recruit Tregs into tumor sites [35]. Overproduction of TGF-β1 in 4T1/TGF-β1 tumor-bearing mice was associated with a higher percentage of CD4^+^CD25^+^Foxp3^+^ cells in the lungs and CD103^+^CD4^+^Foxp3^+^ cells in the spleens, compared with 4T1/RFP tumor-bearing mice and tumor-free mice. Both naringenin and 1D11 treatments significantly reduced the percentage of CD4^+^CD25^+^Foxp3^+^ and CD103^+^CD4^+^Foxp3^+^ cells in the lungs and spleens, respectively, of 4T1/TGF-β1 tumor-bearing mice (*P* <0.05) (Fig. 3a, b).

**Fig. 4:**
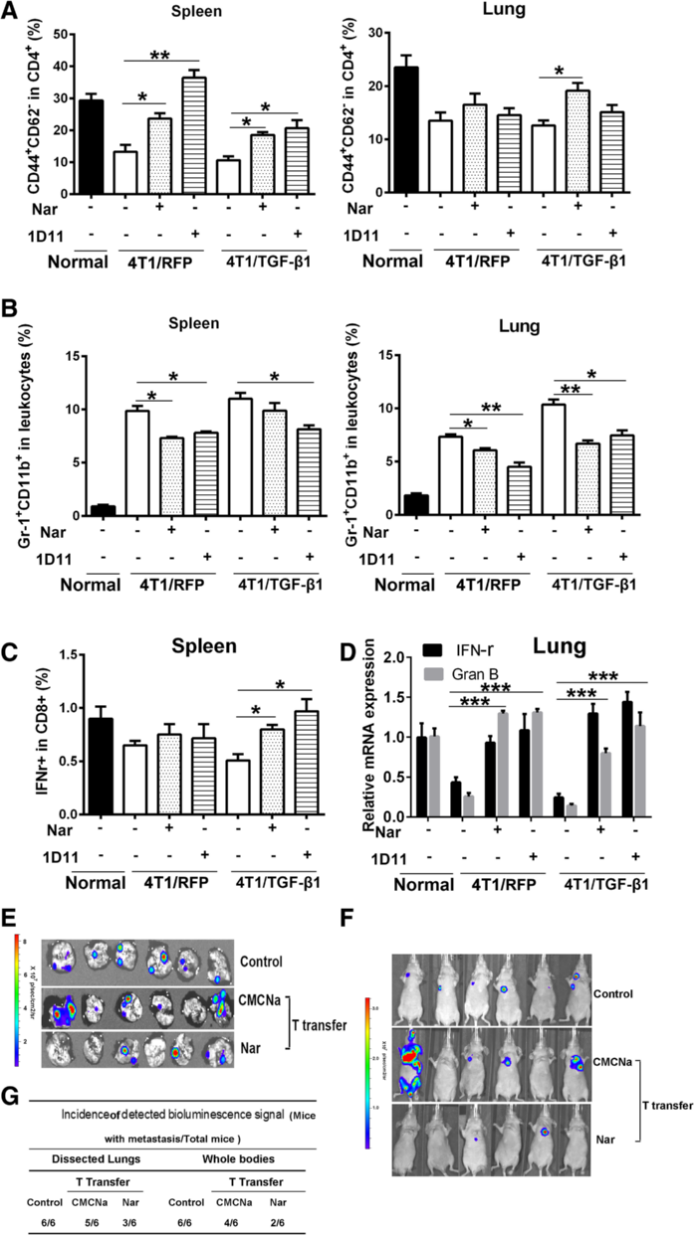
Naringenin (*Nar*) improves the immune activities of T cells. **a** Flow cytometry analysis of T-cell activation marker expressed CD4^+^CD44^+^CD62L^–^ cells in lymphocytes purified from spleen and lung tissues. **b** Flow cytometry analysis of the percentage of Gr-1^+^CD11b^+^ cells (MDSC) in leukocytes purified from spleen and lung tissues. Data are from three mice per group. **c** Analysis of IFNγ^+^ specific CD8^+^ cell percentage in lymphocytes purified from the spleen of mice bearing 4T1/RFP tumors or 4T1/TGF-β1 tumors. **d** Analysis of the mRNA level of *IFNγ* and granzyme-B (*Gran B*) in lung samples of mice bearing 4T1/RFP tumors or 4T1/TGF-β1 tumors. Data are from three experiments. *P <0.05, **P <0.01, ***P <0.001. Error bars indicate SE. **e**, **f** Bioluminescence imaging of the dissected lungs **e** and whole bodies **f** of nude mice after transferring T cells from Balb/c mice treated with or without naringenin for 28 days using the IVIS system (*n* = 6). **g** Incidence of pulmonary metastasis of the dissected lungs and whole bodies (*n* = 6). *CMCNa* sodium carboxyl methyl cellulose, *IFN* interferon, *TGF* transforming growth factor

Several studies have indicated that TGF-β1 can induce an immunosuppressive microenvironment that promotes metastasis both in animal models and human cancer patients [36, 37]. The results from clinical trials indicate that inhibitors of TGF-β signaling may enhance anti-tumor immune responses in patients [38], causing us to wonder whether naringenin could also exhibit anti-tumor immune responses in 4T1/TGF-β1 tumor-bearing mice by reducing TGF-β1 production. As expected, tumor-derived TGF-β1 established a systemic immunosuppressive environment that was represented by an increase of the CD4^+^Gr11b^+^ T-cell population and a decrease of the activated subpopulation of CD4^+^CD44^+^CD62L^–^ T cells in lungs and spleens and CD8^+^IFNγ^+^ T cells in spleens (Fig. 4a–c). In addition, the mRNA levels of effector molecules such as IFNγ and granzyme-B in the lungs were reduced after tumor injection (*P* <0.001) (Fig. 4d). Naringenin treatment effectively reversed the tumor-mediated immunosuppressive environment; its effect was comparable with that of 1D11 treatment (Fig. 4a–d).

To further ascertain whether the enhanced T-cell activity after naringenin treatment facilitates its inhibitory effect on breast cancer pulmonary metastasis, we transferred the T cells from 4T1/TGF-β1 tumor-bearing mice into nude mice along with 4T1/TGF-β1 tumor cells. Compared with nude mice with T cells transferred from normal mice (*Control*) and from the mice without naringenin treatment (*CMCNa*), the nude mice that received T cells from mice with naringenin treatment showed a decreased incidence of pulmonary metastasis (100 % (6/6) vs 83 % (5/6) vs 50 % (3/6) in dissected lungs; and 100 % (6/6) vs 67 % (4/6) vs 33 % (2/6) in whole bodies) (Fig. 4e–g). In addition, one of the nude mice transferred with T cells from mice without naringenin treatment showed expanded whole body metastases besides lung metastases (Fig. 4f).

### Naringenin reduces TGF-β1 secretion and invasion of 4T1 cells in vitro

**Fig. 5:**
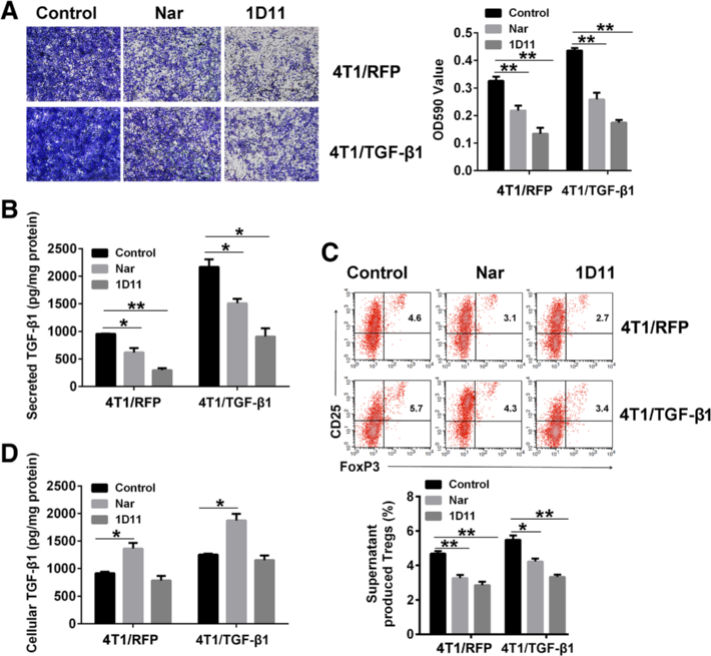
Naringenin (*Nar*) inhibits TGF-β1 secretion and the invasion of 4T1 cells in vitro. **a** Invasion of cultured 4T1/RFP cells (*upper*) and 4T1/TGF-β1 cells (*lower*) after treatment with 100 μM of Nar or 5 μg/ml of 1D11 for 48 hours. The transwell cells were stained and imaged under microscopy (100×) following fixation (*left*). The stained cells were dissolved using DMSO and OD590 values were determined using the multimode reader (*right*). **b** Secreted TGF-β1 concentration in supernatant from 4T1/RFP cells and 4T1/TGF-β1 cells after treatment with Nar or 1D11 for 48 hours. **c** Flow cytometry analysis of the percentage of Tregs induced by the cell supernatants. Percentage gated cells are shown (*upper*). Column charts show the mean percentage obtained from three experiments (*lower*). **d** TGF-β1 concentration in 4T1/RFP cells and 4T1/TGF-β1 cells after treatment with Nar or 1D11 for 48 hours. *P <0.05, **P <0.01, ***P <0.001. Error bars indicate SE. *TGF* transforming growth factor, *Treg* regulatory T cell

Overexpression of *Tgf-β1* in 4T1 cells promoted pulmonary metastasis, and naringenin inhibited pulmonary metastasis by decreasing TGF-β1 levels. We then asked whether naringenin inhibited the secretion of TGF-β1 from 4T1/TGF-β1 cells. Compared with 4T1/RFP cells, the overexpression of *Tgf-β1* in 4T1/TGF-β1 cells led to a significant increase of secreted TGF-β1 (2.3-fold difference, *P* = 0.02) and promoted invasion of the cells (Fig. 5a, b). Knockdown of *Tgf-β1* by TGF-β1 specific small interfering RNA (siRNA) and neutralization of TGF-β1 by 1D11 antibody in 4T1/RFP cells showed an inhibitory effect on the invasion and TGF-β1 secretion of tumor cells (siRNA vs control, 0.46-fold difference, *P* = 0.01; 1D11 vs control, 0.31-fold difference, *P* = 0.003) (Fig. 5a, b and Additional file 6: Figure S4A, B). Naringenin treatment resulted in decreased TGF-β1 secretion (in 4T1/RFP cells, 0.64-fold difference, *P* = 0.04; in 4T1/TGF-β1 cells, 0.69-fold difference, *P* = 0.05) and cell invasion in both 4T1/RFP and 4T1/TGF-β1 cells. The combination of naringenin and TGF-β1 specific siRNA showed no difference in TGF-β1 secretion (*P* = 0.18) and tumor cell invasion (Additional file 6: Figure S4A, B) from each agent alone. These in vitro results provided complementary data to confirm that naringenin suppressed the invasion of breast cancer cells by reducing autocrine TGF-β1 signaling from the tumor cells.

TGF-β1 can convert lymphatic T cells to Tregs. We next investigated whether naringenin could suppress the conversion of Tregs in vitro. In agreement with the in vivo data, the supernatants from cells treated with naringenin or 1D11 showed a suppression of Treg conversion activity (Fig. 5c and Additional file 6: Figure S4C). TGF-β1 siRNA also inhibited the conversion of Tregs in 4T1/RFP cells (0.66-fold difference, *P* = 0.04) (Additional file 6: Figure S4C). Compared with that of control, the supernatant of 4T1/RFP cells treated with TGF-β1 siRNA or 4T1/TGF-β1 cells treated with naringenin, which contained a lower concentration of TGF-β1, showed a reduced capacity to induce the expression of *MMP2* and *MMP9* mRNA in 4T1 cells (Additional file 6: Figure S4D, E). Interestingly, although both naringenin and 1D11 reduced TGF-β1 secretion, 1D11 did not change the intracellular TGF-β1 concentration while naringenin resulted in an intracellular accumulation of TGF-β1 (Fig. 5b, d). The levels of secreted and intracellular TGF-β1 in different human breast cancer cell lines and  293T and MDCK cells treated with naringenin for 48 hours were also detected. The results showed that naringenin significantly decreased TGF-β1 secretion and increased intracellular TGF-β1 concentrations in multiple human breast cancer cells, including MCF7, MDA-MB-231, MDA-MB-436, and MDA-MB-468 cells, but not in 293T and MDCK cells (Additional file 7: Figure S5).

### Naringenin prevents TGF-β1 release

1D11 treatment had no effect on the intracellular TGF-β1 concentration in 4T1 cells, suggesting that naringenin may have a different mechanism of action from siRNA or 1D11 in reducing the secretion of TGF-β1. Thus, we investigated the mechanisms underlying the inhibitory effect of naringenin on TGF-β1 secretion, which involve a series of controlled processes, including transcription, post transcription, and post translation. We surprisingly found that the transcription of *Tgf-β1* was not influenced by naringenin treatment (Additional file 8: Figure S6B). The post-transcriptional regulation of *Tgf-β1* was also investigated using actinomycin D, which blocks mRNA synthesis, in the presence or absence of naringenin. Naringenin did not affect the decay of *Tgf-β1* mRNA in the presence of actinomycin D (*P* = 0.65) (shown in Additional file 6: Figure S4C).

**Fig. 6:**
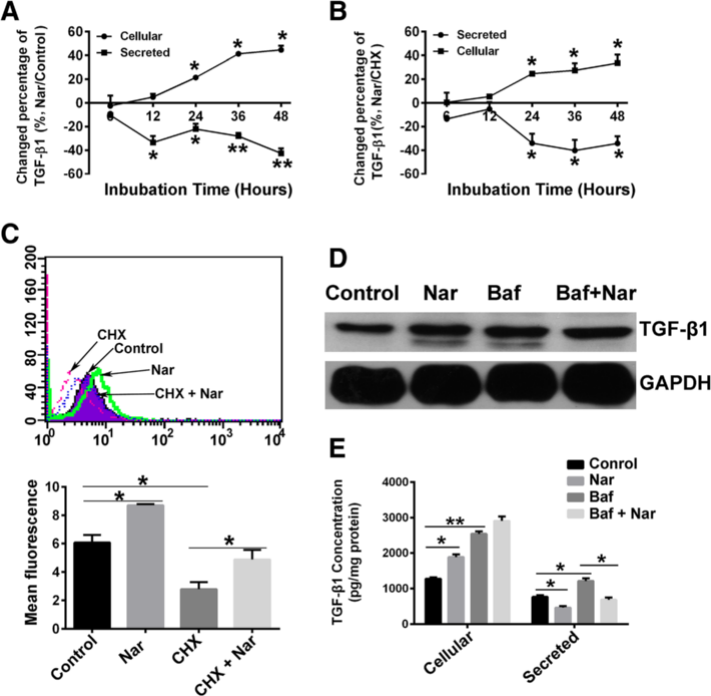
Naringenin (*Nar*) reduces the release of TGF-β1. **a**, **b** Changes of cellular and secreted TGF-β1 in 4T1 cells after treatment with Nar in the absence of CHX (**a** Nar to untreated Control) or the presence of CHX (**b** Nar to CHX treatment) over time. **c** Flow cytometry analysis of the cellular TGF-β1 level in 4T1 cells after treatment with Nar, CHX, or the combination of Nar and CHX for 24 hours. **d** Western blot analysis of the cellular TGF-β1 level in 4T1 cells after treatment with Nar, Bafilomycin A (*Baf*), or the combination of Nar and Baf for 24 hours. **e** Intracellular and secreted TGF-β1 concentrations of 4T1 cells treated with Nar, Baf, or the combination of Nar with Baf for 24 hours were measured by ELISA. *P <0.05, **P <0.01, ***P <0.001. Error bars indicate SE. *CHX* cycloheximide, *GAPDH* glyceraldehyde 3-phosphate dehydrogenase, *TGF* transforming growth factor

Next, we tested whether naringenin regulates the translation of TGF-β1 using cycloheximide (CHX) to block protein synthesis in 4T1 cells. CHX treatment induced a decrease of secreted TGF-β1 and intracellular TGF-β1 (*P* <0.05) (Additional file 9: Figure S7). Naringenin treatment showed that the time-dependent increase of intracellular TGF-β1 was in parallel to a gradual decrease of secreted TGF-β1 into the media (naringenin vs control, from 24 to 48 hours detected, *P* <0.05) (Fig. 6a and Additional file 8: Figure S6). The effects of naringenin on released TGF-β1 and intracellular TGF-β1 were still observed in the presence of CHX (*P* = 0.04 for intracellular TGF-β1; *P* = 0.05 for released TGF-β1 after 48 hours of treatment) (Fig. 6b). Flow cytometry analysis of intracellular TGF-β1 further confirmed that naringenin induced an accumulation of intracellular TGF-β1 in the presence or absence of CHX (in the absence of CHX, 1.4-fold difference, *P* = 0.02; in the presence of CHX, 1.7-fold difference, *P* = 0.04) (Fig. 6c).

TGF-β proteins, synthesized as precursor proteins, are cleaved in the ER and then transported through the TGN to the cell surface for release or to the lysosome for degradation. We therefore hypothesized that naringenin might reduce lysosomal degradation of TGF-β1, resulting in the observed cellular accumulation. To address this hypothesis, we utilized a specific lysosome inhibitor, bafilomycin A1, which prevents protein degradation by raising lysosomal pH. Interestingly, bafilomycin A1 treatment significantly increased the intracellular accumulation of TGF-β1 while also increasing extracellular TGF-β1 release (intracellular, twofold difference, *P* = 0.01; extracellular, 1.6-fold difference, *P* = 0.02), indicating that more TGF-β1 can be released by cells after blocking lysosomal degradation (Fig. 6d, e). In contrast to bafilomycin A1 treatment, naringenin treatment decreased the extracellular release of TGF-β1 even in the presence of bafilomycin A1 (0.57-fold difference, *P* = 0.02) (Fig. 6d, e), suggesting that the lysosomal degradation process does not involve in the effects of naringenin.

### Naringenin inhibits protein kinase C activation and trafficking of TGF-β1

**Fig. 7:**
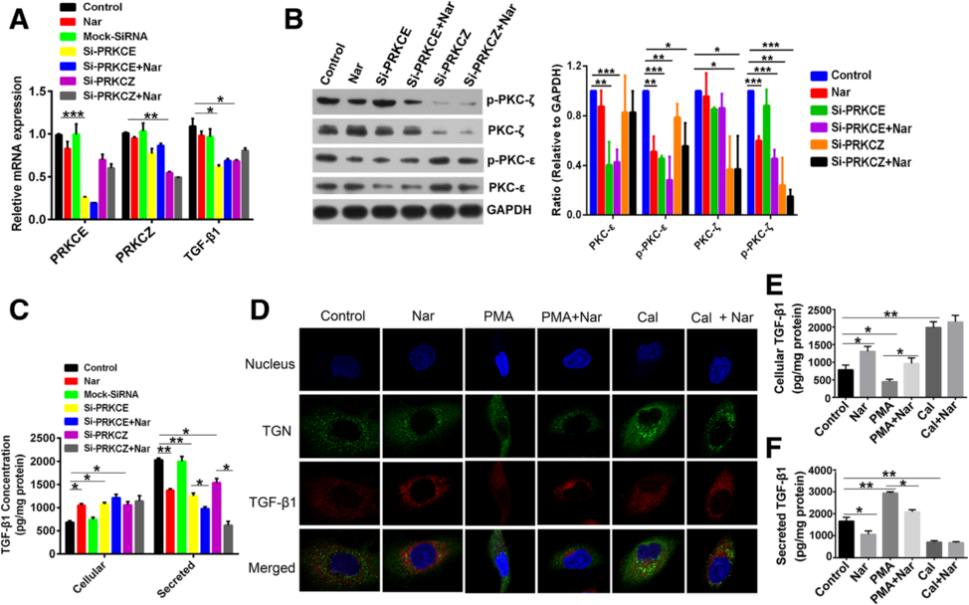
Naringenin (*Nar*) inhibits the activation of PKC-ε and PKC-ζ. **a** Analysis of the relative mRNA level of *PRKCE*, *PRKCZ*, and *Tgf-β1 * in 4T1 cells treated with 100 μM of Nar for 24 hours with or without transfection using the siRNAs of *PRKCE* or *PRKCZ*. **b** Western blot analysis of PKC-ε, p-PKC-ε, PKC-ζ, and p-PKC-ζ expression in 4T1 cells treated with Nar for 48 hours with or without transfection using siRNAs of *PRKCE* or *PRKCZ*. Imaging of western blot bands (*left*) and quantitative data of protein expression relative to GAPDH from three repeats (*right*). **c** Cellular and secreted TGF-β1 concentrations of 4T1 cells treated with Nar for 48 hours with or without transfection using the siRNAs of *PRKCE* or *PRKCZ*. **d** Confocal imaging of TGF-β1 and TGN in 4T1 cells treated with Nar, phorbol 12-myristate 13-acetate (*PMA*), calphostin C (*Cal*), the combination of Nar and PMA, or Nar and Cal for 48 hours. **e**, **f** Cellular **e** and secreted **f** TGF-β1 concentrations of 4T1 cells treated with Nar, PMA, Cal, the combination of Nar and PMA, or Nar and Cal for 48 hours. *P <0.05, **P <0.01, ***P <0.001. Error bars indicate SE. *GAPDH* glyceraldehyde 3-phosphate dehydrogenase, *PKC* protein kinase C, *TGF* transforming growth factor, *PMA* phorbol 12-myristate 13-acetate

Previous studies have demonstrated that the protein kinase C (PKC) family of kinases is involved in the transport and sorting of many proteins from the TGN to cell membranes and organelles [39], prompting us to address whether PKC is involved in the trafficking of TGF-β1. To test this hypothesis, we first investigated the expression of 11 members of the PKC family in 4T1/RFP cells and 4T1/TGF-β1 cells (Additional file 10: Table S2). The results showed that *Tgf-β1* expression positively correlated with *PRKCE* (3.1-fold difference, *P* = 0.008) and *PRKCZ* mRNAs (1.5-fold difference, *P* = 0.04), suggesting that PKC-ε and PKC-ζ proteins might be involved in the transport of TGF-β1. Silencing PKC-ε and PKC-ζ in 4T1 cells using the respective siRNAs showed a decrease of *Tgf-β1* mRNA expression (PKC-ε silencing, 0.57-fold difference, *P* = 0.04; PKC-ζ silencing, 0.63-fold difference, *P* = 0.05) (Fig. 7a). These results suggest that TGF-β1 and PKC-ε or PKC-ζ may work in a positive feedback loop. To further investigate the relationship between TGF-β1 and PKC-ε or PKC-ζ, we determined the production of TGF-β1 protein in 4T1 cells after silencing PKC-ε or PKC-ζ; meanwhile, the phosphorylation status of PKC-ε and PKC-ζ was also detected. Compared with untreated cells, PKC-ε and PKC-ζ siRNA-treated cells showed reduced expressions of the targeted genes, both the total protein levels and the phosphorylation levels (Fig. 7b); correspondingly, the amount of TGF-β1 released from the cells was significantly reduced (PKC-ε silencing, 0.62-fold difference, *P* = 0.008; PKC-ζ silencing, 0.76-fold difference, *P* = 0.038), but resulted in an accumulation of cellular TGF-β1 (PKC-ε silencing, 1.57-fold difference, *P* = 0.015; PKC-ζ silencing, 1.54-fold difference, *P* = 0.04) (Fig. 7c). Silencing PKC-ε or PKC-ζ with siRNA had similar results to naringenin treatment, which decreased secretion of TGF-β1 (0.64-fold difference, *P* = 0.006) while increasing intracellular levels of TGF-β1 (1.54-fold difference, *P* = 0.012) (Fig. 7c). However, naringenin treatment had no effect on the mRNA and protein expression of PKC-ε or PKC-ζ, but did inhibit their phosphorylation (Fig. 7a, b). The combination of naringenin and siRNAs targeting PKC-ε or PKC-ζ exhibited a synergistic effect on decreasing TGF-β1 secretion (Fig. 7c). Taken together, our results suggest that both PKC-ε and PKC-ζ regulate TGF-β1 trafficking, and that naringenin inhibits the activation of PKC-ε and PKC-ζ, which prevents TGF-β1 secretion.

Golgi-bound PKC has been shown to have a crucial impact on vesicle formation at the TGN [39]. Therefore, we sought to address whether the naringenin-mediated cellular accumulation of TGF-β1 is due to blocking the transport of TGF-β1 from the TGN to the plasma membrane. We used phorbol 12-myristate 13-acetate (PMA) to activate PKC in 4T1 cells, and observed the distribution of the TGN and TGF-β1 by immunofluorescence. Compared with untreated cells, PMA-treated cells had an increased density of TGN in the vicinity of the plasma membrane with a more dispersed cytoplasmic distribution and lower fluorescence intensity of TGF-β1, indicating that more TGF-β1 was released from the cells (Fig. 7d). Indeed, we found that PMA treatment resulted in a 0.57-fold decrease of TGF-β1 concentration in the cells and a 1.77-fold increase of TGF-β1 concentration in the culture media (Fig. 7e, f). The addition of calphostin C, a specific inhibitor of PKC, resulted in an increased density of the TGN and TGF-β1-containing vesicles around the nucleus with brighter fluorescence (Fig. 7d), along with more cellular accumulation (2.5-fold difference, *P* = 0.01) and less release of TGF-β1 (0.42-fold difference, *P* = 0.02) (Fig. 7e, f). Cells treated with naringenin showed similar responses to the addition of calphostin C (Fig. 7d–f), suggesting that naringenin is a potential inhibitor of PKC. Furthermore, addition of naringenin to PMA-treated cells partially reversed the effects of PMA, but naringenin had no effect on calphostin C-treated cells (Fig. 7d–f). Taken together, naringenin reduced TGF-β1 secretion by blocking its cellular transport through inhibition of PKC rather than promoting its degradation and/or inhibiting its expression.

## Discussion

In this study, we demonstrated that TGF-β1 derived from breast cancer cells promotes tumor cell metastasis through immune suppression. Similar to 1D11 antibody, which increased the population of anti-tumor effector T cells, naringenin inhibited breast cancer metastasis by blocking TGF-β1 secretion both in vitro and in vivo. Interestingly, as opposed to 1D11, which had no effect on the intracellular TGF-β1 concentration, naringenin induced an accumulation of TGF-β1 in 4T1 murine breast cancer cells as well as multiple human breast cancer cells (Additional file 7: Figure S5). Further investigations into the mechanism revealed that naringenin blocked the intracellular trafficking of TGF-β1 via inhibiting PKC-ε and PKC-ζ phosphorylation rather than modulating the degradation or expression of TGF-β1. This may provide a novel therapeutic strategy toward targeting the TGF-β signaling pathway.

Silencing PKC-ε or PKC-ζ in invasive breast cancer cells inhibited TGF-β1 release, suggesting that the trafficking of TGF-β1 from the TGN compartment to the cell membrane is mediated by PKC proteins. Similar to PKC inhibitor treatment, naringenin treatment resulted in an increased colocalization of TGF-β1 and the TGN in breast cancer cells. These experiments demonstrate that naringenin prevents TGF-β1 transport from the TGN by inhibiting PKC activation (Fig. 7 and Additional file 11: Figure S8). Intriguingly, naringenin showed no effect on TGF-β1 secretion in normal mice [26] or normal cells (Additional file 7: Figure S5), which suggests that naringenin may only have this activity in cells expressing abnormally high TGF-β1 levels. Naringenin reduced TGF-β1 secretion by inhibiting PKC activity, which is a different mechanism of action from the current inhibitors of TGF-β1 signaling, such as TGF-β1 antibodies and antagonists of its receptors, which directly target the pathway. These inhibitors have been shown to have undesired systemic side effects due to the multiple physiological functions of TGF-β1. PKC is a critical regulator of the trafficking required for cytokine secretion; therefore, targeting PKC may provide a previously unidentified avenue for designing therapeutic interventions for multiple cytokine disorders.

Flavonoids have been reported to modulate *Tgf-β1* expression and/or secretion in different contexts. Quercetin has been shown to induce large amounts of TGF-β1 secretion in leukemic blasts, resulting in an inhibition of acute myeloid leukaemia (AML) and acute lymphoid leukemia (ALL) blast growth [40]. However, a recent study demonstrated that quercetin alleviated bile duct ligation-induced *Tgf-β1* expression in rats [41]. Similarly, dihydromyricetin was shown to inhibit proliferation of Hepa l-6 cells by downregulating *Tgf-β1* expression [42]. These differences in the regulation of TGF-β1 by flavonoid compounds may be due to their different chemical structures. Future studies will need to investigate the mechanisms by which different flavonoid compounds selectively regulate TGF-β1 signaling.

TGF-β1 overexpression, which is tumor suppressive in the primary lesion yet promotes metastatic dissemination, has been found in a majority of breast cancer patients [43]. These duel functions of TGF-β1 in cancer development have led to the potential of combining TGF-β1 inhibitors with radiation or peptide vaccine for cancer therapies [44, 45]. We have previously found that naringenin not only downregulated *Smad3* expression and phosphorylation [27, 46] but also inhibited TGF-β1 ligand/receptor binding [47]. In this study, we further found that naringenin prevented TGF-β1 secretion from breast cancer cells by inhibiting PKC phosphorylation. The extensive involvement of naringenin in the TGF-β1 pathway makes it a potentially more potent antagonist of TGF-β signaling than other PKC inhibitors or the TGF-β1 antibody. Given that naringenin can modulate the intracellular trafficking of TGF-β1, we expect that naringenin could effectively prevent tumor metastasis, making it an attractive candidate to be used in combination with other anti-cancer therapies [48].

## Conclusions

We present data demonstrating that naringenin represents an attractive therapeutic candidate for the prevention of breast cancer metastasis, although it has no benefit for the primary tumor. The combination of naringenin with radiation or peptide vaccine might provide new therapeutic strategies to treat cancer progression and metastasis.

### Ethics approval and consent to participate

All animal protocols used for this study were approved by the Institutional Animal Care and Use Committee.

## Additional files

Additional file 1: Presents the supplementary methods. (DOC 54 kb) Additional file 2: Figure S1. Showing the secreted TGF-β1 concentration and OD590 values of transwell cells in different human breast cancer cells. **A** Secreted TGF-β1 concentrations in different cells were measured by ELISA after cultured for 48 hours. **B** Invasion of transwell cells stained and dissolved using DMSO and OD590 values determined using the multimode reader. Error bars indicate SE. (TIF 155 kb) Additional file 3: Figure S2. Showing the effect of naringenin (*Nar*) on the growth of primary 4T1 tumors. **A** Bioluminescence imaging of pulmonary metastasis in vivo. Mice bearing 4T1 tumors were treated with Nar or 1D11 24 days and then were imaged with bags to avoid the bioluminescence from primary tumor. **B** Bioluminescence imaging of primary tumors in vivo. Mice bearing tumors were treated with Nar or 1D11 for 24 and 34 days and then were imaged the bioluminescence using the IVIS system. **C** Volume of primary breast tumor measured by calipers. Tumor volumes were measured by calipers after tumor injection from 10 to 34 days. Error bars indicate SE. (TIF 1908 kb) Additional file 4: Table S1. Presenting the incidence of pulmonary metastasis (mice with metastasis/total mice). Tumor-bearing mice treated with naringenin or 1D11 were imaged on day 24 using bags to avoid the bioluminescence from primary tumor. The mice with pulmonary metastases were numbered based on the bioluminescence signal. (TIF 26 kb) Additional file 5: Figure S3. Showing the effects of naringenin (*Nar*) on the expression of TGF-β1 and Foxp3 in vivo. **A**, **B** TGF-β1 concentrations in the homogenates of tumor, spleen, lung, and serum from mice after treatment for 14 and 28 days. Mice bearing 4T1/RFP tumors or 4T1/TGF-β1 tumors were treated with Nar for 14 and 28 days. The tissues were homogenated and collected for TGF-β1 detection by ELISA kit. **C** Immunohistochemical analysis of TGF-β1 expression and location in lung tissue sections of mice bearing 4T1/RFP tumors or 4T1/TGF-β1 tumors after treatment with Nar for 14 and 28 days. **D** Expression of TGF-β1 and Foxp3 proteins in lung tissues of mice bearing 4T1/RFP tumors or 4T1/TGF-β1 tumors after treatment with Nar for 14 and 28 days by western blot analysis. **P* <0.05, ***P* <0.01, ****P* <0.001. Error bars indicate SE. (TIF 1071 kb) Additional file 6: Figure S4. Showing the effects of naringenin (*Nar*) on the invasion of and the TGF-β1secretion of 4T1 cells. **A** Invasion of cultured 4T1/RFP cells, 4T1/TGF-β1 cells, and 4T1 cells knocked down by TGF-β1siRNAwith or without Nar treatment for 48 hours (*upper*). Invasion of 4T1 cells cultured with the individual supernatants from the upper cells for 48 hours (*lower*). **B** Measurement of the secreted TGF-β1 concentration in supernatants from 4T1/RFP cells, 4T1/TGF-β1 cells, and 4T1 cells knocked down by TGF-β1 siRNA with or without Nar treatment for 48 hours. **C**. Flow cytometry analysis of the percentage of Tregs induced by the supernatants. The lymphocytes were purified from spleen tissues of 8-week-old mice. The supernatants from 4T1/RFP cells, 4T1/TGF-β1 cells, and 4T1 cells knocked down by TGF-β1 siRNA with or without Nar treatment for 48 hours were used to culture the lymphocytes which activated with monoclonal antibodies against CD3/CD28. After 72 hours of incubation, the lymphocytes were collected and stained with CD4^+^CD25^+^Foxp3^+^ antibodies for flow cytometry analysis. **D**, **E** Analysis of mRNA levels of *MMP2* and *MMP9* in 4T1 cells treated with the supernatants from 4T1/RFP cells, 4T1/TGF-β1 cells, and 4T1 cells knocked down by TGF-β1 siRNA with or without Nar treatment for 48 hours. **P* <0.05, ***P* <0.01, ****P* <0.001. Error bars indicate SE. (TIF 4025 kb) Additional file 7: Figure S5. Showing the effects of naringenin (*Nar*) on the cellular and secreted TGF-β1 concentration in different cell lines. **A** Cellular TGF-β1 concentrations in cultured cells after the treatment of 100 μM naringenin for 48 hours. **B** Secreted TGF-β1 concentrations in the media of different cultured cells with 100 μM naringenin treatment for 48 hours. The levels of TGF-β1 concentrations were determined by ELISA. Data are from at least three independent experiments. **P* <0.05, ***P* <0.01, ****P* <0.001. Error bars indicate SE. (TIF 128 kb) Additional file 8: Figure S6. Showing the effects of naringenin (*Nar*) on the transcription and decay of *Tgf-β1 * mRNA. **A** Secreted TGF-β1 concentrations in the media of cultured 4T1 cells with naringenin treatment for different times. **B** Analysis of mRNA levels of *Tgf-β1 * in cultured 4T1 cells with or without naringenin treatment for 24 and 48 hours. **C** Analysis of the stability of *Tgf-β1* mRNA in 4T1 cells after naringenin treatment. Cells were pretreated with 100 μM naringenin or diluent for 2 hours, followed by addition of 10 μg/ml actinomycin D to inhibit the synthesis of new RNA. The levels of remained TGF-β1 mRNA were determined by qPCR. Data are from at least three independent experiments. **P* <0.05, ***P* <0.01, ****P* <0.001. Error bars indicate SE. (TIF 139 kb) Additional file 9: Figure S7. Showing the changed percentages of the cellular and the secreted TGF-β1 of 4T1 cells after treatment with 10 μM cycloheximide (CHX treatment to untreated control) over time. The intracellular and the secreted TGF-β1 proteins were analyzed using ELISA kit. (TIF 48 kb) Additional file 10: Table S2. Presenting the mRNA expression copies of *PKC* family genes in 4T1/RFP cells and 4T1/TGF-β1 cells. Total RNA was extracted and real-time qPCR was performed for detection the relative mRNA expression copies of *PKC* to 10^6^ of *GAPDH* copies. **P* <0.05, ***P* <0.01. (TIF 36 kb) Additional file 11: Figure S8. Showing western blot analysis of the expression and activation of PKC proteins in lung tissues of mice bearing 4T1/RFP tumors or 4T1/TGF-β1 tumors after naringenin (*Nar*) administration for 28 days (detailed procedure is described in Methods). (TIF 63 kb)

## Abbreviations

CHX: Cycloheximide
CMCNa: Sodium carboxyl methyl cellulose
ELISA: Enzyme-linked immunosorbent assay
EMT: Epithelial to mesenchymal transition
ER: Endoplasmic reticulum
IFNγ: Interferon gamma
IQ: Interquartile
MDSC: Myeloid-derived suppressor cells
Nar: Naringenin
PKC: Protein kinase C
qPCR: Quantitative PCR
SE: Standard error
siRNA: Small interfering RNA
TGF: Transforming growth factor
TGN: Trans-Golgi network
Treg: Regulatory T cell
