# Supplementary material for: Naringenin prevents TGF-β1 secretion from breast cancer and suppresses pulmonary metastasis by inhibiting PKC activation
Source: Breast Cancer Res. 2016 Apr 1;18:38. doi: 10.1186/s13058-016-0698-0 (PMC4818388; doi:10.1186/s13058-016-0698-0)
Supplement: Additional file 1: — Presents the supplementary methods. (DOC 54 kb) [file 13058_2016_698_MOESM1_ESM.doc]

**Additional file 1: Supplementary Methods**

***In vivo* Breast Cancer Metastasis Experiment**

Four-week-old female Balb/c mice were purchased from Weitonglihua Tech. (Beijing, China) and housed in the Animal Care Facility of the Institute of Biophysics, CAS, China. All animal protocols used for this study were approved by the Institutional Animal Care and Use Committee. 2 X 10^4^ of the vector control (4T1/RFP) or TGF-β1 over-expressing 4T1-Luc2 cells (4T1/TGF-β1) were injected into the forth fat pad of Balb/c mice. To compare the potential of lung metastasis between 4T1/RFP cells and 4T1/TGF-β1 cells, the tumors-bearing mice were injected intraperitoneally with 150 mg/kg luciferin and the lung areas were imaged using IVIS system (Spectrum, Xenogen). To avoid the bioluminescence from primary tumor, the primary tumors were wrapped up with light-proof bags. Four weeks after the primary tumors growth, mice were sacrificed after i.p. injection of luciferin for 15 min and lungs were collected for imaging or weighting to determine the amount of metastases. The primary tumors were also isolated and weighted.

To determine the therapy effect of naringenin on breast tumor growth and metastasis, 140 mice (18 to 20 mice per each group, 11 to 13 mice used for immune T cells detection and 7 mice used for tumor growth and metastasis imaging and survival analysis) were divided into 7 groups. The tumor-free group was administrated with PBS/CMCNa. The mice bearing 4T1/RFP tumor or 4T1/TGF-β1 tumor were divided into three groups, the one group was administrated with PBS/CMCNa, the second group was oral gavaged with 200 mg/kg of naringenin daily for 30 days after tumor implantation and the third group was injected intraperitoneally with 5 mg/kg of 1D11 antibodies once one week for three weeks after tumor injection. Before optical imaging, the mice were injected intraperitoneally with 150 mg/kg luciferin for 15 min and the whole body and the lung areas were imaged bioluminescence using IVIS system at day 24 and 34 after treatment. The lymphocytes were purified from spleen and lung tissues after 28 days treatment for T cell immune activation detection. At day 34 after treatment, TGF-β1 concentration in lung homogenates and serum were detected by ELISA. At day 14 and 28 after treatment of naringnein, the lung tissues were divided into three portions for the isolation of RNA, for cell lysate production, and for making paraffin blocks for immunohistochemistry. After sacrificing the mice, the primary tumors, spleens, lungs and serum were collected and homogenated to product lysates. The activated TGF-β1 levels in the lysates and serum were detected by ELISA.

**Immunohistochemistry**

Immunohistochemical studies were done in 5-µm sections of paraffin-embedded lung tissues using antibodies for TGF-β1 to determine its content in the lung materials. The slides were incubated in citrate buffer for 20 min in a steamer and endogenous peroxidase was blocked by incubation with 3% H_2_O_2_ for 20 min at room temperature. The anti-TGF-β1 polyclonal antibodies were used in a dilution of 1:200. The slides were then stained with the secondary antibody in a dilution of 1:500. To determine the protein expression, stained slides were examined under fluorescence microscopy.

**Transfer of T cells and 4T1/TGF-β1 Cancer Cells into Nude Mice**

T cells were isolated from the spleen of normal mice or 4T1/TGF-β1 tumor-bearing Balb/c mice (each group of three mice). Balb/c mice were injected 20,000 of 4T1/TGF-β1 tumor cells per mouse on the forth fat pad. 200 mg/kg of naringenin or 1% CMCNa as control was gavaged to the mice on the same day after tumor cell injection. The spleens were isolated from normal mice and tumor-bearing mice at day 14 after tumor cells injection, minced into small pieces, and then placed into ACK hypotonic lysis solution (Sigma, USA) at room temperature for 5 min to lyse red blood cells (RBCs). Following lysis, the remaining cells were filtered through 70-µm meshes (BD Inc.) and washed with HBSS buffer for three times. T cells were isolated using a pan T cell isolation kit (MiltenyiBiotech, Germany) in accordance with the manufacturer's protocol.

Six-week-old female Balb/c nude mice were purchased from Weitonglihua Tech. (Beijing, China). The nude mice were divided into three groups (six mice per group) each mouse received 5,000 of 4T1/TGF-β1 tumor cells through i.v. injection, meanwhile, 500,000 of T cells from normal mice (the first group, control group), tumor-bearing mice administrated with 1% CMCNa (the second group, CMCNa group), and tumor-bearing mice administrated with naringenin (the third group, Nar group), were injected intravenously into the nude mice. T cells with tumor cells were transferred once a week for three weeks. To evaluate the antitumor effect of the adoptive T cell transfer, we detected the bioluminescence signal from lung metastasis using *in vivo* optical imaging. The mice were injected intraperitoneally with 150 mg/kg luciferin for 15 min and the whole bodies were imaged by bioluminescence using IVIS system at day 28 after tumor cells injection. Following this observation, these mice were sacrificed, and the lungs were dissected and imaged by bioluminescence.

**TGF-β1 ELISA Assay**

For quantification of TGF-β1 *in vitro*, cells after different treatments were incubated in 6-well culture plates in X-Vivo 15 medium without FBS. After incubation for different times, the media were collected and passed through filters with pore diameter sizes of 0.45 µm. Total protein of cells was extracted as described in Western blot assay section. Before analysis, total proteins in cells and in the media were treated with HCl to convert all mature TGF-β1 into a form recognized by the antibody used in the assay. TGF-β1 protein levels were then determined using the TGF-β1 E_max_ Immunoassay system (Promega, USA). The operational approach was performed according to manufacture specification.

For quantification of TGF-β1 *in vivo*, the primary tumors, spleens, lungs and serum were collected and homogenated to product lysates after the mice were sacrificed. The lysates were diluted and the activated TGF-β1 protein levels were detected by the TGF-β1 E_max_ Immunoassay system (Promega, USA).

**Knocking Down of Specific Genes with siRNA**

Small interfering RNAs (siRNAs) respectively against TGF-β1 (Invitrogen), PKCE and PKCZ (Santa Cruz), and the mock siRNA (negative control) were used to knock down the specific genes. Cells at 80% confluence were transfected with siRNAs by lipofectimine 2000 reagent (Invitrogen). After 24 h transfection, cells were treated with or without naringenin (100 μM) for 48 h. For transwell assay, the supernatants were collected and added in the chambers with transwell filters. For TGF-β1 quantification, the lysis of treated cells and the supernatants were analyzed using the TGF-β1 ELISA kit (Promega). For real-time PCR assay, treated cells were collected, and total mRNA was isolated for RT-PCR assay with gene-specific primers.

**Real-time RT-PCR (qPCR) Assay**

Total RNAs were isolated from 4T1 cells（Control lines，TGF-β1 overexpression lines or TGF-β1 silencing lines）treated with or without naringenin (100 μM) using Trizol (Invitrogen, CA, USA). For detection of the mRNA expression in lung tissues, total RNA were isolated from fresh lung tissues homogenates by Trizol. The mRNAs were reversely transcribed to cDNAs by M-MLV reverse transcriptase (Invitrogen, CA, USA). qPCR was performed using SYBR Green qPCRSuperMix (invitrogen) according with the instrument to detect the expression of TGF-β1, MMP2, MMP9, IFNr, Gran B, PKCE and PKCZ mRNA levels. Melting curve analysis referred to instrument documentation. Gene expression was normalized to GAPDH expression and presented as fold-change compared to the control experiments for all samples. All assays were performed at least three times and the results from one experiment were provided.

**The *in vitro* Cell Invasion Assay**

Cell invasion assay was performed using 24-well BD Falcon^TM^Cell culture insert (8 µm pore size, Becton Dickinson USA). Briefly, the solubilized basement matrigel (BD Tech.) was added onto the top chamber to generate a membrane. Cells were trypsinized and resuspended in X-vivo 15 medium and seeded at a density of 5×10^4^ cells per well onto the top chamber. The bottom chamber was filled with X-vivo 15 medium contained with samples (control of 0.1% DMSO, 5 µg/mL 1D11 or 100 μM of naringenin) as a chemic attractant. After 48 h treatment, the filter membrane were fixed with cool methanol and stained with crystal violet. The non-invading cells in the upside of filters were carefully removed with a cotton swab, and the cells on the lower surface of the filters were shooted under a Leica pathological image and analytical system (Germany). The stained cells were dissolved using 500 µL of DMSO and OD_590_ were determined using the Multimode Reader. The experiments were performed in triplicate and repeated three times.

**Quantification of Intracellular TGF-β1 Using Flow Cytomytry**

4T1 cells were treated with vehicle (DMSO), naringenin (100 µM), Cycloheximide (CHX) or CHX combined with naringenin for 24 h. Cells were collected , fixed and permeabilized in fixation/permeabilization buffer (eBioscience), and then stained with TGF-β1 primary antibody. After stained with FITC-conjugated second antibody, the cells were then detected for quantization of fluorescence by a flow cytometer with excitation/emission: 488/519 nm.

**Western Blot Analysis**

The treated 4T1 cells *in vitro* or lung homogenates of mice were lysed with RIPA lysis and extraction buffer (Pierce Biotech, USA). The denatured lysates were separated on a 10% polyacrylamide gel. Protein was transferred onto a polyvinylidenedifluoride membrane. The membrane was blocked using 5% non fat milk and probed for 2 hours at room temperature with primary antibodies for TGF-β1, Foxp3, PKC-ε, p-PKC-ε, PKCς, p- PKCς and GAPDH (Cell Signal). After that, the membranes were washed and probed with a 1:2,000 dilution of peroxidase-conjugated secondary antibodies, and then detected by enhanced chemiluminescence (Amersham Life Sciences, Amersham, UK).

**Confocal Imaging of the Cellular Localization of TGF-β1**

4T1 cells were grown overnight on glass cover slips. Cells were treated with vehicle (DMSO), naringenin (100 µM), PMA (100 nM), PMA with Nar, Calphostin C (Cal) （20 nM）or Cal combination with naringenin for 48 h. Cells were washed and fixed in 4% paraformaldehyde in PBS for 15 min at 37 °C. Samples were permeabilized 0.2% Triton-X 100 in PBS for 15 min and blocked with 1% bovine serum albumin in HBSS for 1h at room temperature. The samples were incubated with the monoclonal goat anti-TGF-β1 (Santa Cruz.) and rabbit anti-TGN46 primary antibody (Abcam) in blocking solution at a concentration of 1 μg/ml. After washing with HBSS, anti-goat IgG conjugated with Alexa Fluor 633 and anti-rabbit IgG conjugated with Alexa Fluor 488 antibodies (Invitrogen Co.) were added in blocking solution to a final concentration of 2 µg/mL. 10 µM Hoechst 333342 (Sigma Chemical Co.) was added for 30 min to visualize nuclei after washing with HBSS. Cells were observed TGF-β1 expression, trans-Golgi network and nuclei using confocal microscopy.
