# Supplementary figures and images for: Naringenin prevents TGF-β1 secretion from breast cancer and suppresses pulmonary metastasis by inhibiting PKC activation
Source: Breast Cancer Res. 2016 Apr 1;18:38. doi: 10.1186/s13058-016-0698-0 (PMC4818388; doi:10.1186/s13058-016-0698-0)

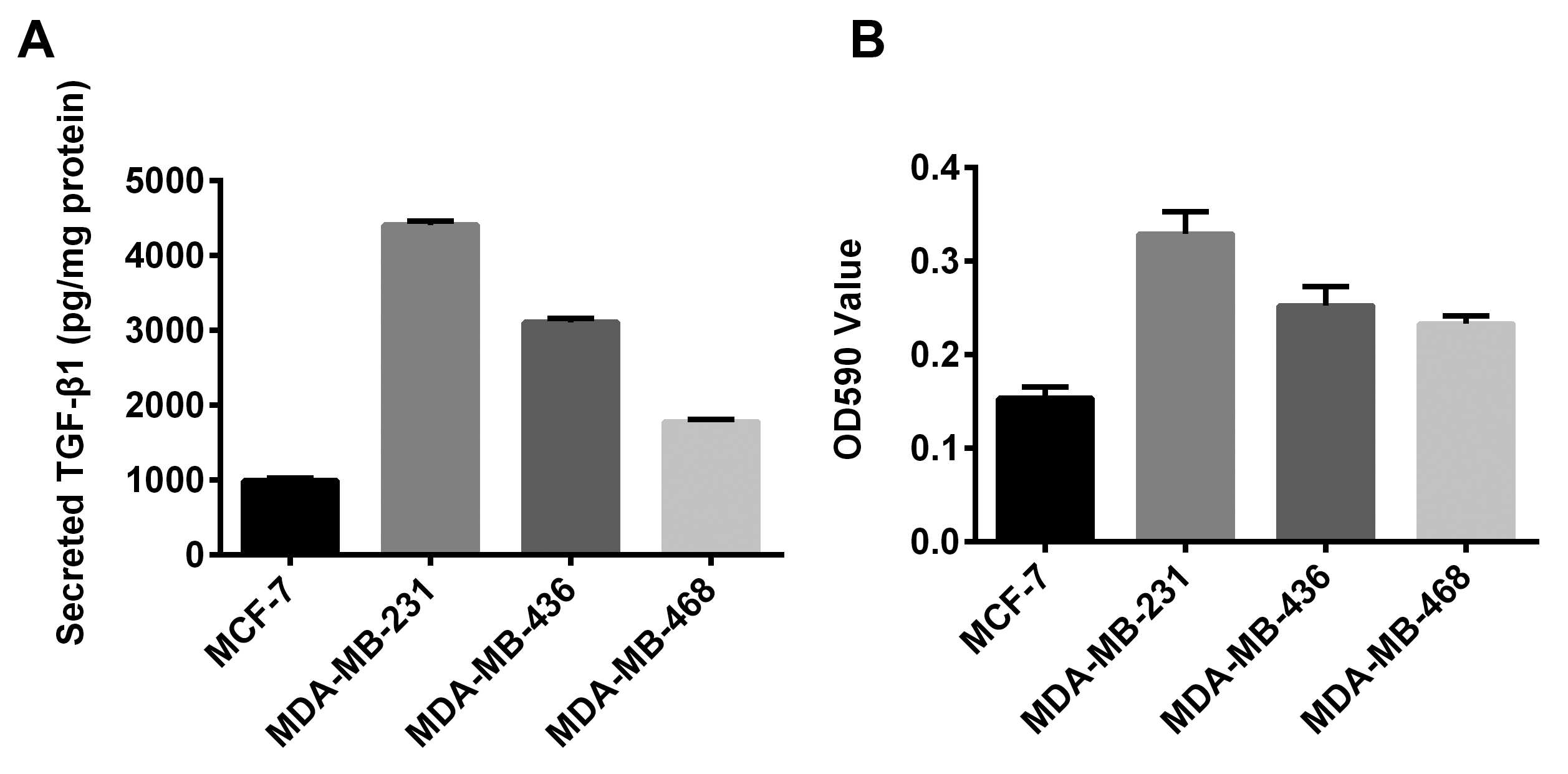

Supplement: Additional file 2: Figure S1. — Showing the secreted TGF-β1 concentration and OD590 values of transwell cells in different human breast cancer cells. A Secreted TGF-β1 concentrations in different cells were measured by ELISA after cultured for 48 hours. B Invasion of transwell cells stained and dissolved using DMSO and OD590 values determined using the multimode reader. Error bars indicate SE. (TIF 155 kb) [file 13058_2016_698_MOESM2_ESM.tif]

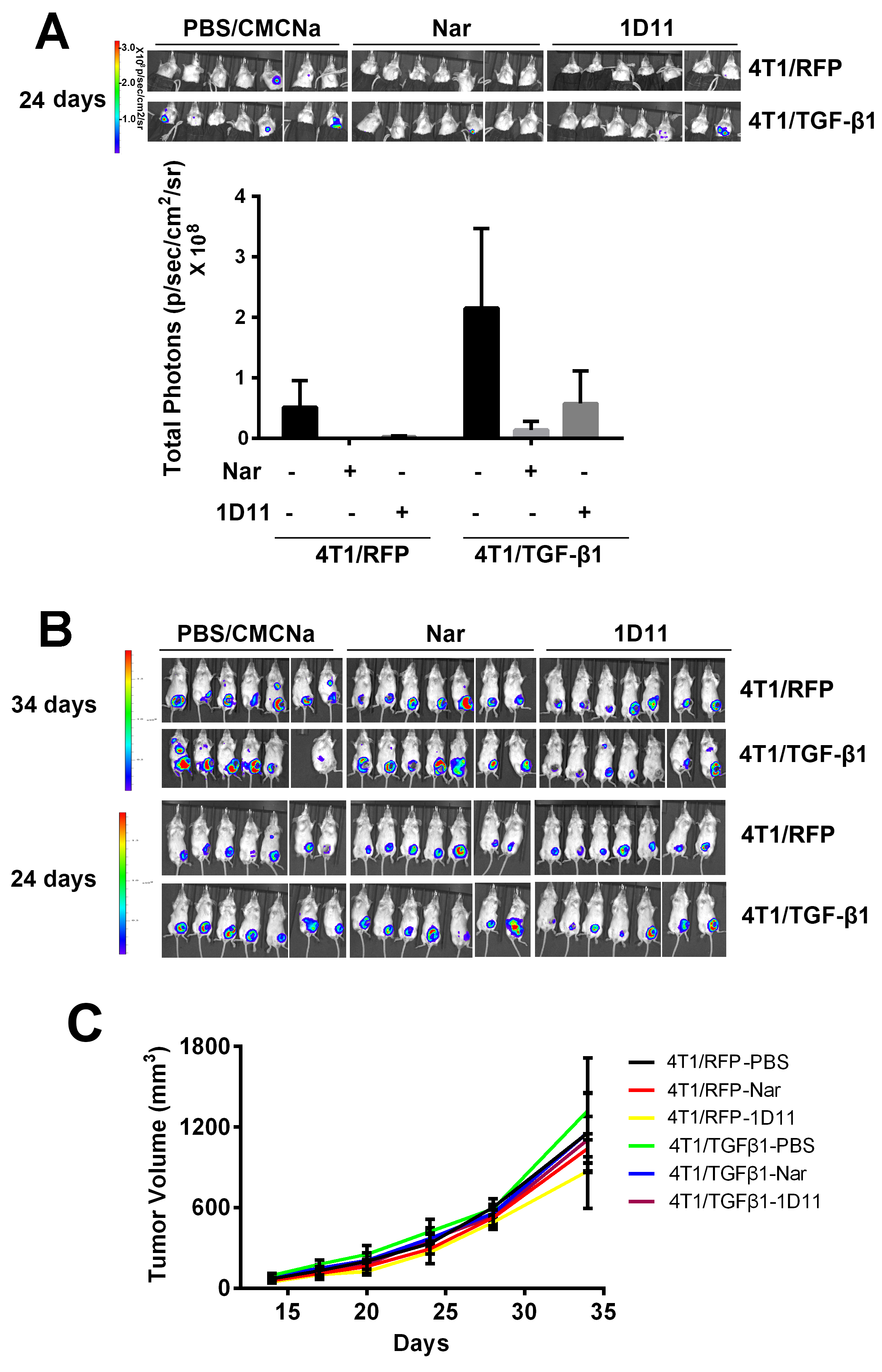

Supplement: Additional file 3: Figure S2. — Showing the effect of naringenin (Nar) on the growth of primary 4T1 tumors. A Bioluminescence imaging of pulmonary metastasis in vivo. Mice bearing 4T1 tumors were treated with Nar or 1D11 24 days and then were imaged with bags to avoid the bioluminescence from primary tumor. B Bioluminescence imaging of primary tumors in vivo. Mice bearing tumors were treated with Nar or 1D11 for 24 and 34 days and then were imaged the bioluminescence using the IVIS system. C Volume of primary breast tumor measured by calipers. Tumor volumes were measured by calipers after tumor injection from 10 to 34 days. Error bars indicate SE. (TIF 1908 kb) [file 13058_2016_698_MOESM3_ESM.tif]

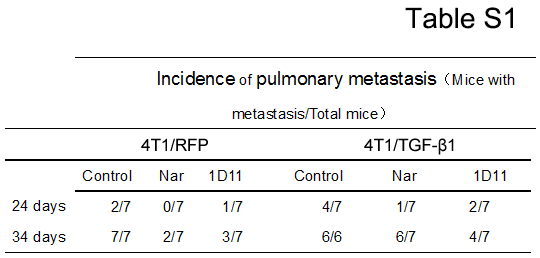

Supplement: Additional file 4: Table S1. — Presenting the incidence of pulmonary metastasis (mice with metastasis/total mice). Tumor-bearing mice treated with naringenin or 1D11 were imaged on day 24 using bags to avoid the bioluminescence from primary tumor. The mice with pulmonary metastases were numbered based on the bioluminescence signal. (TIF 26 kb) [file 13058_2016_698_MOESM4_ESM.tif]

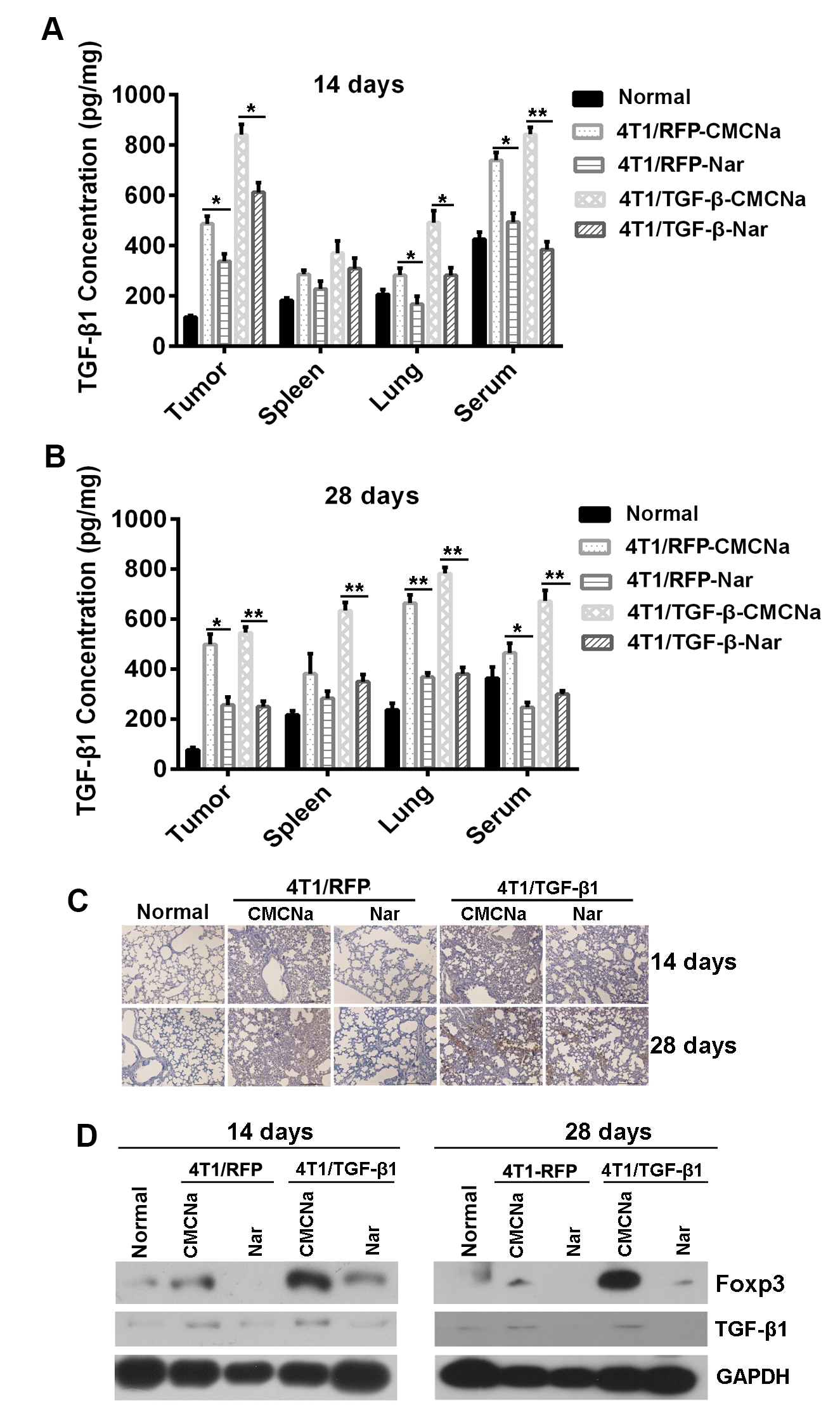

Supplement: Additional file 5: Figure S3. — Showing the effects of naringenin (Nar) on the expression of TGF-β1 and Foxp3 in vivo. A, B TGF-β1 concentrations in the homogenates of tumor, spleen, lung, and serum from mice after treatment for 14 and 28 days. Mice bearing 4T1/RFP tumors or 4T1/TGF-β1 tumors were treated with Nar for 14 and 28 days. The tissues were homogenated and collected for TGF-β1 detection by ELISA kit. C Immunohistochemical analysis of TGF-β1 expression and location in lung tissue sections of mice bearing 4T1/RFP tumors or 4T1/TGF-β1 tumors after treatment with Nar for 14 and 28 days. D Expression of TGF-β1 and Foxp3 proteins in lung tissues of mice bearing 4T1/RFP tumors or 4T1/TGF-β1 tumors after treatment with Nar for 14 and 28 days by western blot analysis. *P <0.05, **P <0.01, ***P <0.001. Error bars indicate SE. (TIF 1071 kb) [file 13058_2016_698_MOESM5_ESM.tif]

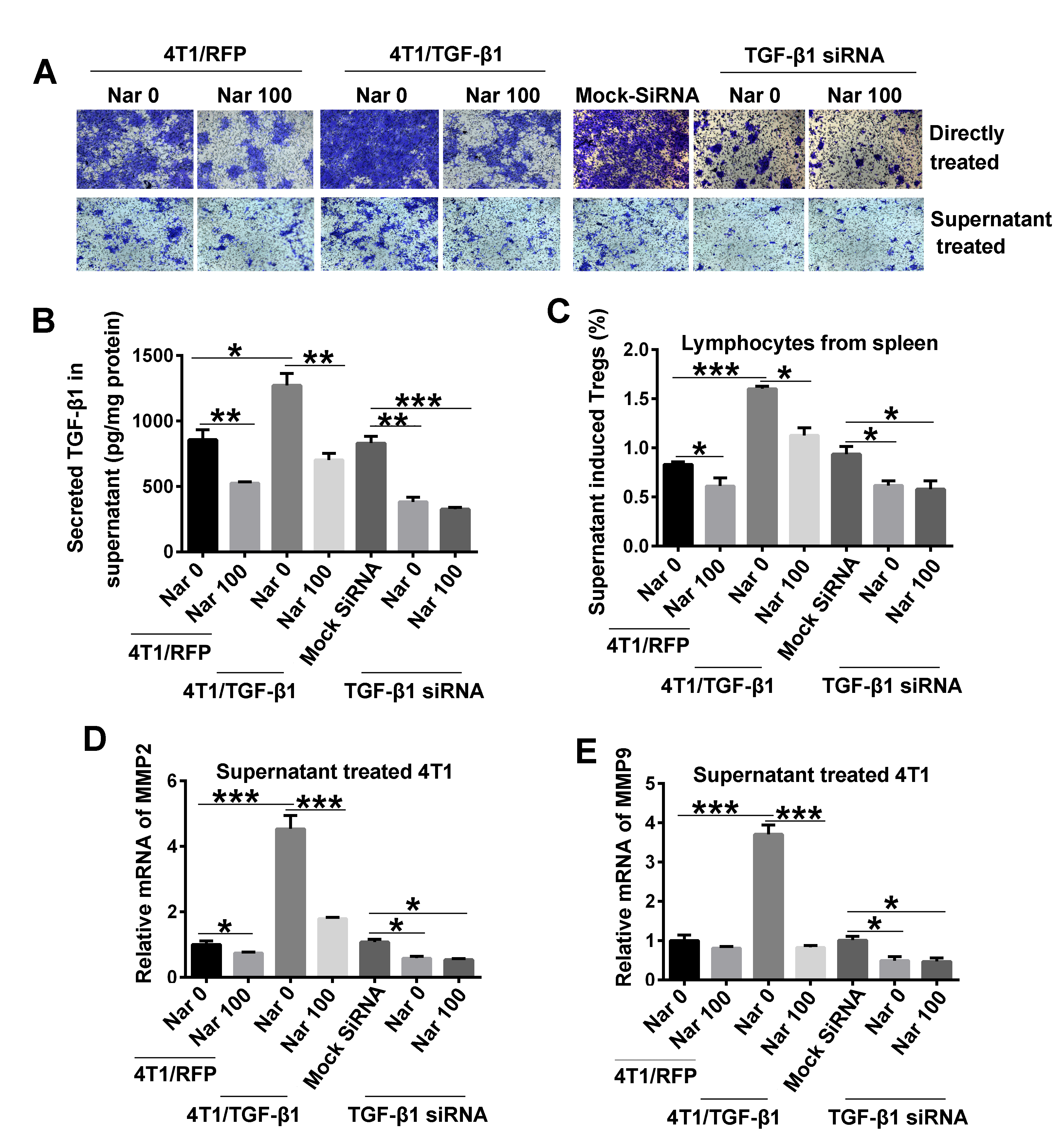

Supplement: Additional file 6: Figure S4. — Showing the effects of naringenin (Nar) on the invasion of and the TGF-β1secretion of 4T1 cells. A Invasion of cultured 4T1/RFP cells, 4T1/TGF-β1 cells, and 4T1 cells knocked down by TGF-β1siRNAwith or without Nar treatment for 48 hours (upper). Invasion of 4T1 cells cultured with the individual supernatants from the upper cells for 48 hours (lower). B Measurement of the secreted TGF-β1 concentration in supernatants from 4T1/RFP cells, 4T1/TGF-β1 cells, and 4T1 cells knocked down by TGF-β1 siRNA with or without Nar treatment for 48 hours. C. Flow cytometry analysis of the percentage of Tregs induced by the supernatants. The lymphocytes were purified from spleen tissues of 8-week-old mice. The supernatants from 4T1/RFP cells, 4T1/TGF-β1 cells, and 4T1 cells knocked down by TGF-β1 siRNA with or without Nar treatment for 48 hours were used to culture the lymphocytes which activated with monoclonal antibodies against CD3/CD28. After 72 hours of incubation, the lymphocytes were collected and stained with CD4+CD25+Foxp3+ antibodies for flow cytometry analysis. D, E Analysis of mRNA levels of MMP2 and MMP9 in 4T1 cells treated with the supernatants from 4T1/RFP cells, 4T1/TGF-β1 cells, and 4T1 cells knocked down by TGF-β1 siRNA with or without Nar treatment for 48 hours. *P <0.05, **P <0.01, ***P <0.001. Error bars indicate SE. (TIF 4025 kb) [file 13058_2016_698_MOESM6_ESM.tif]

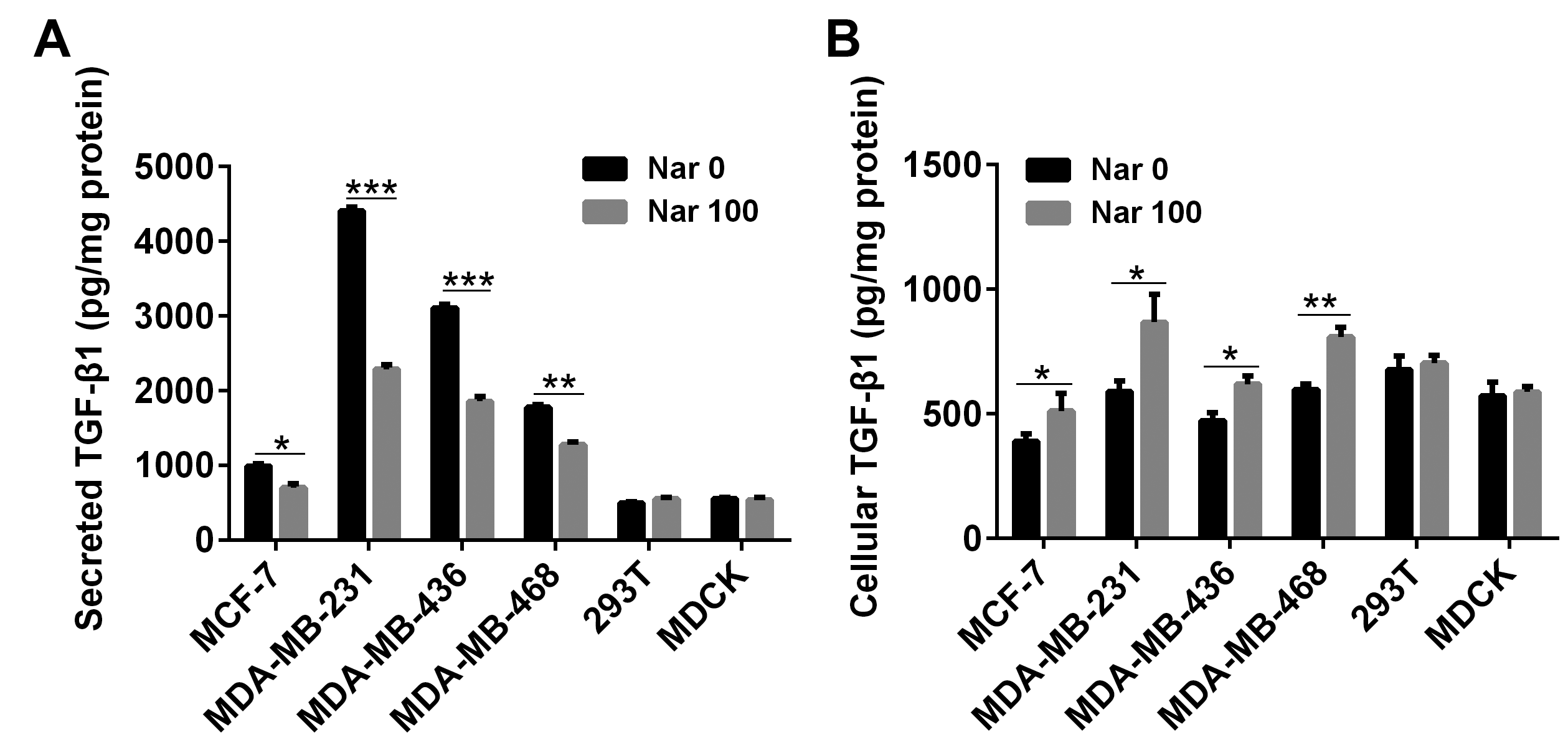

Supplement: Additional file 7: Figure S5. — Showing the effects of naringenin (Nar) on the cellular and secreted TGF-β1 concentration in different cell lines. A Cellular TGF-β1 concentrations in cultured cells after the treatment of 100 μM naringenin for 48 hours. B Secreted TGF-β1 concentrations in the media of different cultured cells with 100 μM naringenin treatment for 48 hours. The levels of TGF-β1 concentrations were determined by ELISA. Data are from at least three independent experiments. *P <0.05, **P <0.01, ***P <0.001. Error bars indicate SE. (TIF 128 kb) [file 13058_2016_698_MOESM7_ESM.tif]

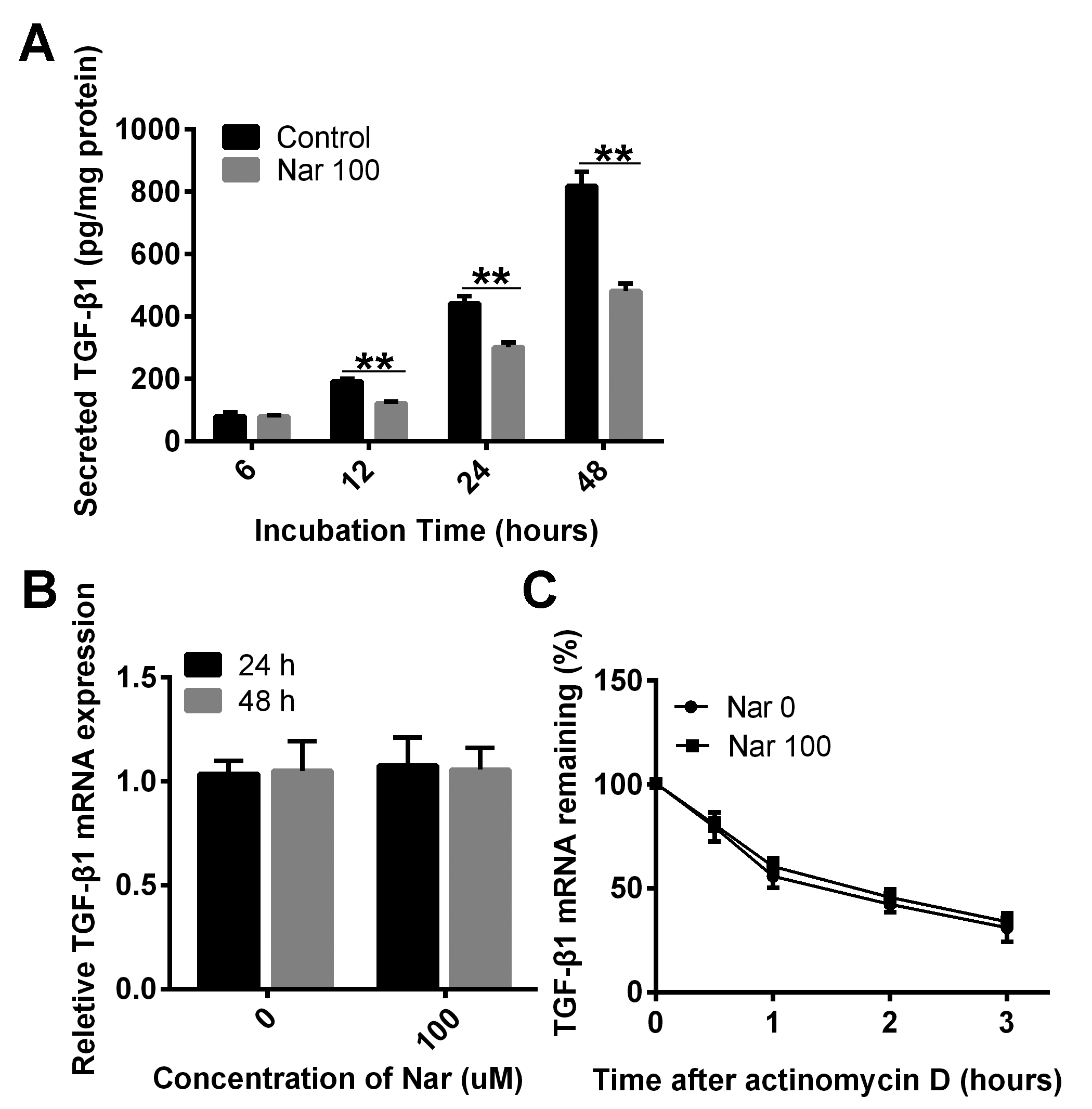

Supplement: Additional file 8: Figure S6. — Showing the effects of naringenin (Nar) on the transcription and decay of Tgf-β1 mRNA. A Secreted TGF-β1 concentrations in the media of cultured 4T1 cells with naringenin treatment for different times. B Analysis of mRNA levels of Tgf-β1 in cultured 4T1 cells with or without naringenin treatment for 24 and 48 hours. C Analysis of the stability of Tgf-β1 mRNA in 4T1 cells after naringenin treatment. Cells were pretreated with 100 μM naringenin or diluent for 2 hours, followed by addition of 10 μg/ml actinomycin D to inhibit the synthesis of new RNA. The levels of remained TGF-β1 mRNA were determined by qPCR. Data are from at least three independent experiments. *P <0.05, **P <0.01, ***P <0.001. Error bars indicate SE. (TIF 139 kb) [file 13058_2016_698_MOESM8_ESM.tif]

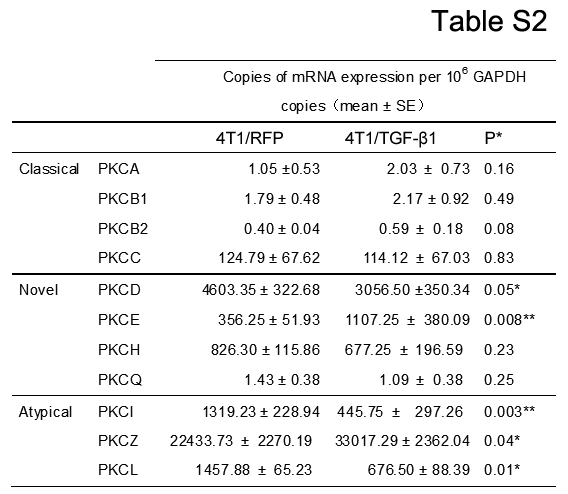

Supplement: Additional file 10: Table S2. — Presenting the mRNA expression copies of PKC family genes in 4T1/RFP cells and 4T1/TGF-β1 cells. Total RNA was extracted and real-time qPCR was performed for detection the relative mRNA expression copies of PKC to 106 of GAPDH copies. *P <0.05, **P <0.01. (TIF 36 kb) [file 13058_2016_698_MOESM10_ESM.tif]

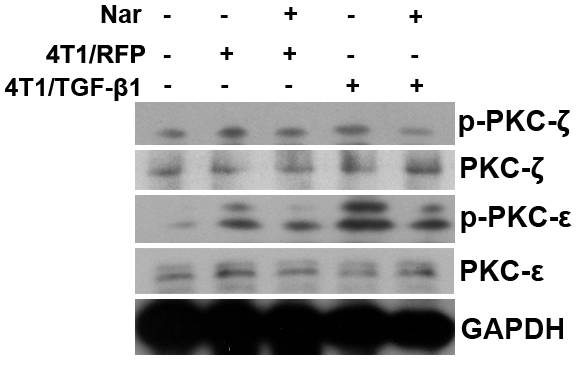

Supplement: Additional file 11: Figure S8. — Showing western blot analysis of the expression and activation of PKC proteins in lung tissues of mice bearing 4T1/RFP tumors or 4T1/TGF-β1 tumors after naringenin (Nar) administration for 28 days (detailed procedure is described in Methods). (TIF 63 kb) [file 13058_2016_698_MOESM11_ESM.tif]
